# Regulation of Egg Quality by Methionine Selenium: Physicochemical and Structural Characteristics of EggShell, Egg Yolk, and Egg White

**DOI:** 10.3390/foods15162859

**Published:** 2026-08-17

**Authors:** Dan Chen, Yaotong Liu, Shiwen Xu

**Affiliations:** 1 College of Veterinary Medicine, Northeast Agricultural University, No. 600 Changjiang Road, Xiangfang, Harbin 150030, China; 2 College of Food Science and Engineering, Northeast Agricultural University, Harbin 150030, China

**Keywords:** methionine selenium, egg quality, lipidomics, FTIR spectroscopy, Cryo-SEM

## Abstract

Selenium is an essential trace element in the body, and supplementing it in production can improve egg quality. Sixty healthy 48-week-old Hy-Line Brown laying hens were randomly assigned to two groups. In this study, the effects of dietary selenomethionine (SeMet) supplementation on the multi-dimensional characteristics of eggshell, egg white, and egg yolk were investigated, including their mechanical properties, mineral distribution, amino acid composition, microstructure, secondary structure, fatty acid composition, and lipidomic profiles. The results showed that compared with the control group (C-group), the air chamber height and egg shape index of the SeMet group (Se-group) were decreased by 21.51% and 1.54%, respectively, after 42 days of feeding. The Haugh units, eggshell strength, and eggshell color of the Se-group were increased by 10.21%, 29.24%, and 31.26%, respectively. This work leverages advanced analytical techniques for systematic sample characterization, including Fourier-transform infrared (FTIR) spectroscopy, cryogenic scanning electron microscopy (Cryo-SEM), and high-resolution tandem mass spectrometry-based lipidomics. Adding SeMet can increase the essential amino acid content and the total content of saturated and unsaturated fatty acids in egg yolk and egg white, thereby maintaining excellent protein patterns and reducing lipid hydrolysis in egg yolk. However, the addition of SeMet did not significantly affect the protein secondary structure or microstructure of egg yolk and egg white.

## 1. Introduction

Selenium is one of the essential trace elements necessary for both human and animal health and plays a vital role in maintaining a healthy condition. There are already several selenium-rich functional foods based on microbes, plants, and animals on the market to help consumers achieve the dietary standards for selenium intake [1,2]. Eggs are an excellent choice for this selenium-rich food because they are both healthy and have a high selenium deposition rate. They are also still a traditional food with reasonable prices in most countries [3]. The method of producing selenium-rich eggs mostly involves adding different selenium sources to the diet and then transferring and depositing selenium into the eggs through the hen’s metabolic activity [4]. The selenium sources in feed include inorganic or organic selenium, such as sodium selenite (SeNa), yeast selenium (SeY), nano selenium (SeN), selenium sulfite (SeS), methionine selenide (SeMet), and hydroxy-selenomethionine (HMSeBA) [5,6,7].

Adding selenium to the diet can effectively increase selenium deposition in the whole egg, which is closely related to factors such as the content, source, and feeding time of selenium in the diet [8,9]. Research has shown that adding different levels of SeY to feed can increase the selenium content of eggs, enhance the antioxidant capacity of laying hens, and extend the retention time of selenium [10]. Han et al. compared the effects of SeNa and SeY. The results showed that adding SeNa and SeY improved the antioxidant capacity of laying hens. Compared with only adding SeY with the same content, adding SeNa and SeY was more cost-effective [11]. Lu et al. found that selenide glucose increased the selenium content, glutathione peroxidase activity, and total antioxidant capacity in egg white and egg yolk, without affecting egg production quality [12]. Yu et al. found that adding 0.1, 0.25, and 0.55 mg/kg SeMet to feed can increase the hatchability of fertile eggs by 13.49%, 3.47%, and 2.3%, respectively; adding 0.1, 0.25, 0.40, and 0.55 mg/kg SeMet can increase the hatching rate of hatchability of hatching eggs by 35.31%, 29.82%, 13.54%, and 12.01%, respectively; adding 0.1, 0.25, 0.40, and 0.55 mg/kg SeMet can increase the fertility of hatching eggs by 13.80%, 24.78%, 12.56%, and 9.89% [13]. Wang et al. found that dietary selenium supplementation improved egg quality—including eggshell thickness, protein height, and Haugh units—in a dose-dependent manner, with the most significant improvements observed at a dosage of 0.9 mg/kg Se [14]. Therefore, incorporating different forms and dosages of selenium into the diet can effectively increase selenium deposition in eggs, improve egg quality, and have a positive impact on egg hatching.

Selenium-rich eggs have become one of the most popular selenium-rich foods due to a variety of unique benefits, including quick production cycles, simple procedures, low costs, and inexpensive product pricing. At present, research on selenium-rich eggs mainly focuses on how to improve selenium content, the distribution and existing forms of selenium, and the bioavailability of selenium. However, comprehensive investigations into the mechanical properties, microstructure, secondary structure, and nutritional composition of different egg fractions (eggshell, albumen, and yolk) remain limited. Therefore, adding SeMet to the diet of laying hens and monitoring the changes in egg quality, eggshell, egg white, and egg yolk during the 42-day feeding period can enrich basic research on selenium-rich eggs, provide theoretical support for anti-selenium-rich technology breeding, and contribute new active ingredients for the development and application of selenium-rich eggs for food, health products, and medicine.

## 2. Materials and Methods

### 2.1. Materials and Reagents

Sixty healthy 48-week-old Hy-Line Brown laying hens were numbered 1–60 and randomly assigned to two groups by random number drawing: the control group (C-group, basal diet) and the SeMet group (Se-group, basal diet supplemented with 0.75 mg/kg SeMet). Hens were fed their respective experimental diets for 42 days. Each group consisted of three replicates of ten hens, with each replicate serving as an experimental unit. The total sample size was sixty hens; replicates were the experimental units, and individual hens were the sampling units for statistical analyses. The primary outcome measure for sample size calculation was laying rate. The basic diet consisted of 40% concentrated feed and 60% corn. The composition and nutritional levels of concentrated feed are listed in Table 1. During the feeding period, 16 h of light per day and an indoor temperature between 20~23 °C were maintained. Laying hens could freely drink and eat. Conventional vaccination procedures were used. All laying hens maintained good health during the feeding period. On days 7, 14, 21, 28, 35, and 42, 10 eggs were randomly collected in duplicate units and marked with group and date for measurement. All animal operations complied with the requirements of the Guidelines for Care and Use of Laboratory Animals of Northeast Agricultural University. All experiments were approved by the Animal Ethics Committee of Northeast Agricultural University (SRM-11). The project identification code is NEAUEC20240315.

### 2.2. Production Performance Measurements of Laying Hens

The number of eggs laid and broken eggs and the total egg weight (g) were recorded every day during feeding. The average egg weight, laying rate, and egg production were calculated.

$$\mathrm{Egg}\;\mathrm{production}\;(g/\mathrm{piece}/\mathrm{day})=\frac{\mathrm{total}\;\mathrm{egg}\;\mathrm{weight}\;\mathrm{per}\;\mathrm{replicate}\;\mathrm{per}\;\mathrm{day}}{\mathrm{number}\;\mathrm{of}\;\mathrm{laying}\;\mathrm{hen}}$$

$$\mathrm{Average}\;\mathrm{egg}\;\mathrm{weight}\;(g)=\frac{\mathrm{total}\;\mathrm{egg}\;\mathrm{weight}}{\mathrm{number}\;\mathrm{of}\;\mathrm{eggs}\;\mathrm{laid}}$$

$$\mathrm{Laying}\;\mathrm{rate}\;(\%)=\frac{\mathrm{number}\;\mathrm{of}\;\mathrm{eggs}\;\mathrm{laid}}{\mathrm{number}\;\mathrm{of}\;\mathrm{chickens}}\times 100$$

### 2.3. Egg Quality Measurements

A TPA texture analyzer (TA.new plus, ISENSO, New York, NY, USA) combined with an A/ETK egg testing device (*n* = 10) was used to measure egg weight, egg yolk membrane strength, egg yolk height, and egg yolk width. A digital Vernier caliper was used to measure the yolk diameter and calculate the score in Haugh units.

### 2.4. Mineral Content Measurements

Inductively coupled plasma–mass spectrometry (7800, Agilent, Santa Clara, CA, USA) was used to determine the mineral content in eggshell, egg white, and egg yolk. The eggshell was dried after removing the eggshell film at room temperature. A total of 0.5 g from the center area of each eggshell (0.5 g of egg yolk or egg white freeze-dried powder) was crushed into powder before passing it through a 40-mesh sieve, and then placed in a digestion tube for microwave digestion. After removing the acid, the sample was transferred into a flask and diluted with deionized water to 50 mL. Element levels were analyzed using three samples from each group.

### 2.5. Physical and Mechanical Property Measurements

Digital Vernier calipers (500-151-30, Mitutoyo, Kawasaki, Japan) were used to measure the diameter and height of the eggs and the air chamber and to calculate the egg shape index. The eggshell strength, thickness, and weight were measured using a TPA texture analyzer combined with an A/ETK egg testing device. The eggshell color was measured by a colorimeter (EMT7300-II, Robotmation, Tokyo, Japan).

### 2.6. Amino Acid Measurements

The hydrolyzed amino acid content in egg yolk or egg white was determined according to the method of Liu et al. [15]. First, 0.08 g of egg yolk freeze-dried powder sample was weighed (0.04 g of egg white freeze-dried powder sample) and added to 10.0 mL of 6 mol/L hydrochloric acid. Nitrogen was blown for 10 min to replace the air in the liquid, and acid hydrolysis was performed in a vacuum-sealed hydrolysis bottle at 110 °C for 22 h. The sample was then transferred to a volumetric flask to a constant volume of 50 mL before removing 1 mL and passing it through a 0.22 μm filter twice. The content of hydrolyzed amino acids was analyzed using an amino acid analyzer (L-8900, Hitachi, Ltd., Tokyo, Japan).

The free amino acid content in egg yolk or egg white was determined according to the method of Qing et al. [16]. An amount of 5 g of the sample was dissolved in 35 mL of deionized water, then 5 mL was taken from it and mixed with 7 mL of 5% trichloroacetic acid. After overnight incubation at 4 °C and centrifugation at 12,000× *g* at 4 °C for 30 min, the supernatant was collected and passed through a 0.2 μm filter.

### 2.7. Fourier Transform Infrared (FTIR) Spectroscopy Measurements

An appropriate amount of eggshell powder sample was mixed with potassium bromide before compression. An FTIR spectrometer (Nicolet iS 10, Thermo Fisher Scientific Co., Ltd., Waltham, MA, USA) was used to scan the entire wavelength range from 4000 to 400 cm^−1^, with a scanning resolution of 4 cm^−1^ and 32 scans. Baseline calibration was performed by subtracting the water background. An appropriate amount of freeze-dried egg yolk or egg white powder was placed on the ATR attachment of the FTIR spectrometer. The measurement conditions were the same as above.

### 2.8. Scanning Electron Microscopy (SEM) Measurements

The microstructure of the eggshells was determined by SEM (SU8010; Hitachi, Tokyo, Japan). After 42 days of feeding, three eggshells were chosen at random from the C-group and Se-group to measure the microstructure of the eggshells and remove the contents inside the eggshells. Two eggshell samples (approximately 1 cm^2^/piece) from the equatorial region of each egg were collected, boiled for 10 min in a 2% NaOH solution, and dried at room temperature. These samples were observed under a 3.0 kV field emission scanning electron microscope.

### 2.9. Cryo Scanning Electron Microscopy Measurements (Cryo-SEM)

The microstructure of egg yolk and egg white was evaluated using Cryo-SEM (Hitachi, Tokyo, Japan). The mixture was sublimated at −90 °C for 15 min and sprayed with gold. Then, the sample was observed at −140 °C. The microscope was operated at an accelerating voltage of 5 kV, with a beam current of 10 mA.

### 2.10. Fatty Acid Content of Egg Yolk Measurements

Egg yolk and chloroform-methanol solution (*v*/*v*, 2:1) were mixed at a ratio of 1:10 and then subjected to ultrasonic treatment for 1 h. The egg yolk was shaken in a gas bath at 25 °C for 30 min, filtered by suction, placed in a separatory funnel, and then left to stand; the lower chloroform layer was removed after clarification. Nitrogen was blown until the solvent completely evaporated. The extracted oil was placed in a brown bottle and frozen at −20 °C for later use. Methylation treatment of egg yolk lipids was performed with reference to GB 5009.168-2016 (China, 2016) [17].

The fatty acids in egg yolk were determined using headspace purge capture triple quadrupole gas chromatography–mass spectrometry (8890-7000D, Agilent, Santa Clara, CA, USA). The chromatographic column was a DB-5 MS; the injection port temperature was 280 °C; the initial column temperature was 50 °C, held for 3 min, then programmed to 220 °C at a rate of 10 °C/min and held for 15 min. The temperature was then programmed at a rate of 5 °C/min to 230 °C, with a retention time of 5 min. The carrier gas was high-purity nitrogen with a flow rate of 1 mL/min. The split ratio was 1:50, and the injection volume was 1 μL. The temperature of the fourth-stage rod was 150 °C, the temperature of the transmitter was 250 °C, the temperature of the ion source was 230 °C, and the scanning range was 50–550 Da.

### 2.11. Lipidomics Measurements in Egg Yolk

One milligram of egg yolk oil was weighed, dissolved in 1 mL of a chloroform-methanol (*v*:*v* = 2:1) mixed solution, passed through a 0.22 μm membrane, and stored in an injection bottle. A four-stage rod time-of-flight tandem mass spectrometry detector (AB SCIEX TRIPIETOF 5600, AB SCIEX, Framingham, MA, USA) was used to detect lipidomics.

Chromatographic detection conditions: Chromatographic column with model ACQUITY UPLC BEH C18 (100 mm × 2.1 mm, 1.7 μm); column temperature 60 °C; injection volume, 2 μL; flow rate, 0.4 mL/min; detection time, 20 min; elution procedure, 0–0.5 min, 20% B; 0.5–1.5 min, 20% B-40% B; 1.5–3 min, 40–60% B; 3–15 min, 60–98% B; 15–5.1 min, 98–20% B; 15.1–20 min, 20% B. Mass spectrometry detection conditions: Positive-ion detection mode, and primary and secondary mass spectrometry scanning ranges of 500–1700 m/z and 50–1700 m/z, respectively. Each gas path uses nitrogen gas. The specific parameters are detailed in Table 2.

### 2.12. Statistical Analysis

The analysis values are expressed as the means ± SDs. One-way analysis of variance (ANOVA) was used for the statistical analysis in the SPSS 19.0 program. The mean differences were calculated using Duncan’s multiple-range tests for means with a 95% confidence limit (*p* < 0.05).

## 3. Results

### 3.1. Changes in Production Performance and Egg Quality

The results of studying the effect of adding SeMet to feed on production performance showed that the laying rate in the C-group was 0.83 ± 0.01%, while that in the Se-group was significantly higher at 0.88 ± 0.01% (*p* < 0.05). The breaking rate in the C-group was 0.03 ± 0.01%, while that in the Se-group was also significantly higher at 0.01 ± 0.01% (*p* < 0.05). The pass rate in the C-group was 0.88 ± 0.05%, while that in the Se-group was significantly higher at 0.93 ± 0.02% (*p* < 0.05). The average egg weight in the C-group was 61.52 ± 1.18 g, while the average egg weight in the Se-group was 63.11 ± 1.84 g (Table S1).

After 42 days of feeding, the Se-group exhibited a 21.51% reduction in air chamber height relative to the C-group (*p* < 0.05, Table 3). While protein height, Haugh units, and yolk index showed increases in the Se-group, these differences were not statistically significant at the terminal time point. Notably, air chamber height was also significantly lower in the Se-group at day 28 (*p* < 0.05), aligning with the overall trend of improved egg freshness retention. These results indicate that dietary SeMet selectively enhances specific egg quality traits linked to storage stability.

### 3.2. Changes in Mineral Element Content of Eggs

**Figure 1:**
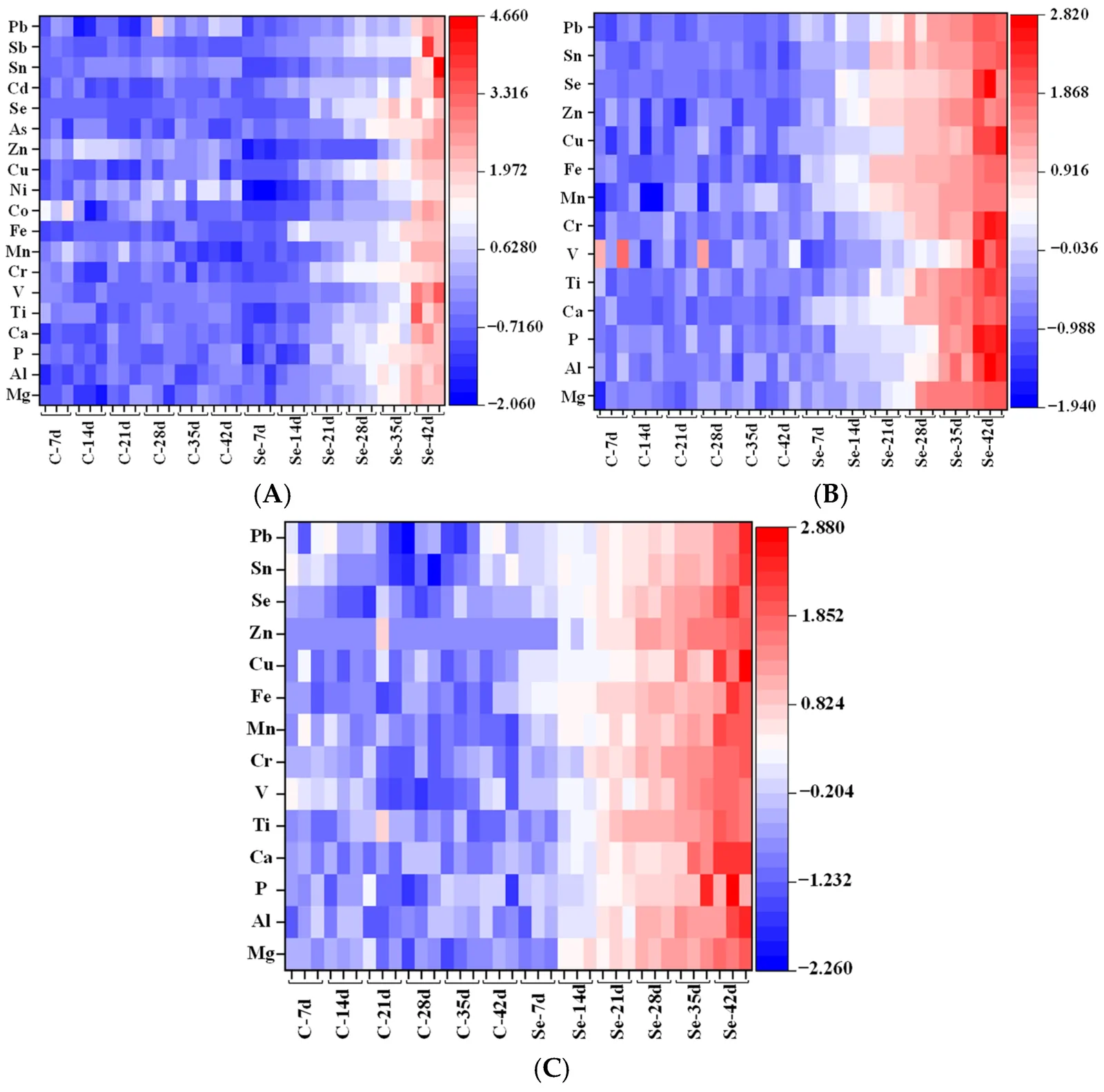
Changes in mineral element distribution (eggshell (**A**), egg yolk (**B**), and egg white (**C**)) during the 42-day SeMet supplementation period. Mineral concentrations were quantified using inductively coupled plasma-mass spectrometry (ICP-MS, Agilent 7800, Agilent Technologies, Santa Clara, CA, USA) after microwave digestion and acid removal, followed by dilution to 50 mL with deionized water.

The heatmap of the detection results of 18 mineral elements in the eggshells of the C-group and Se-group is shown in Figure 1A. Compared with the C-group, the content of most elements in the eggshells of the Se-group showed an upward trend with the increase in feeding days, The content of Sb, Sn, Se, Zn, Cu, Co, Mn, Ca, and P elements contents in the eggshell of eggs fed for 42 days significantly increased (*p* < 0.05), while the content of Fe, V, Ni, As, and Al significantly decreased (*p* < 0.05).

The heatmaps of the content detection results of 14 mineral elements in the egg yolk of the C-group and Se-group are shown in Figure 1B. Compared with the C-group, the content of Se in the collected egg yolks of the Se-group after 14 days of feeding significantly increased (*p* < 0.05), while that of Sn, Zn, Cu, Fe, Cr, Ca, and P significantly decreased (*p* < 0.05).

The heatmaps of the detection results of 14 mineral elements in the egg white of the C-group and Se-group are shown in Figure 1C. The levels of Se and Sn in the egg white collected from the Se-group at 21 days of feeding were significantly higher than (*p* < 0.05) those in the C-group, but the levels of Sb, Cu, V, Ti, and Al were significantly lower than (*p* < 0.05) those in the C-group.

### 3.3. Changes in Physicochemical and Structural Properties of Eggshells

#### 3.3.1. Changes in Physical and Mechanical Properties of Eggshell

The quality of the eggshell is a crucial factor in the production of laying hens, and a thicker eggshell is better for transporting eggs. The relationship between the changes in eggshell thickness, egg shape index, eggshell strength, eggshell weight, and eggshell color and the feeding days of hen eggs is shown in Table 4. There were no significant changes in the eggshell thickness, eggshell strength, or egg shape index of the C-group or Se-group with increasing feeding days. However, the color of eggshells in both groups gradually darkened with the increase in feeding days. Compared with the C-group, the Se-group showed a significant increase in eggshell strength after 42 days of feeding (*p* < 0.05). Meanwhile, the eggshell color of the Se-group was significantly higher than that of the C-group after feeding for 14 and 42 days (*p* < 0.05).

#### 3.3.2. Changes in FTIR of Eggshell

**Figure 2:**
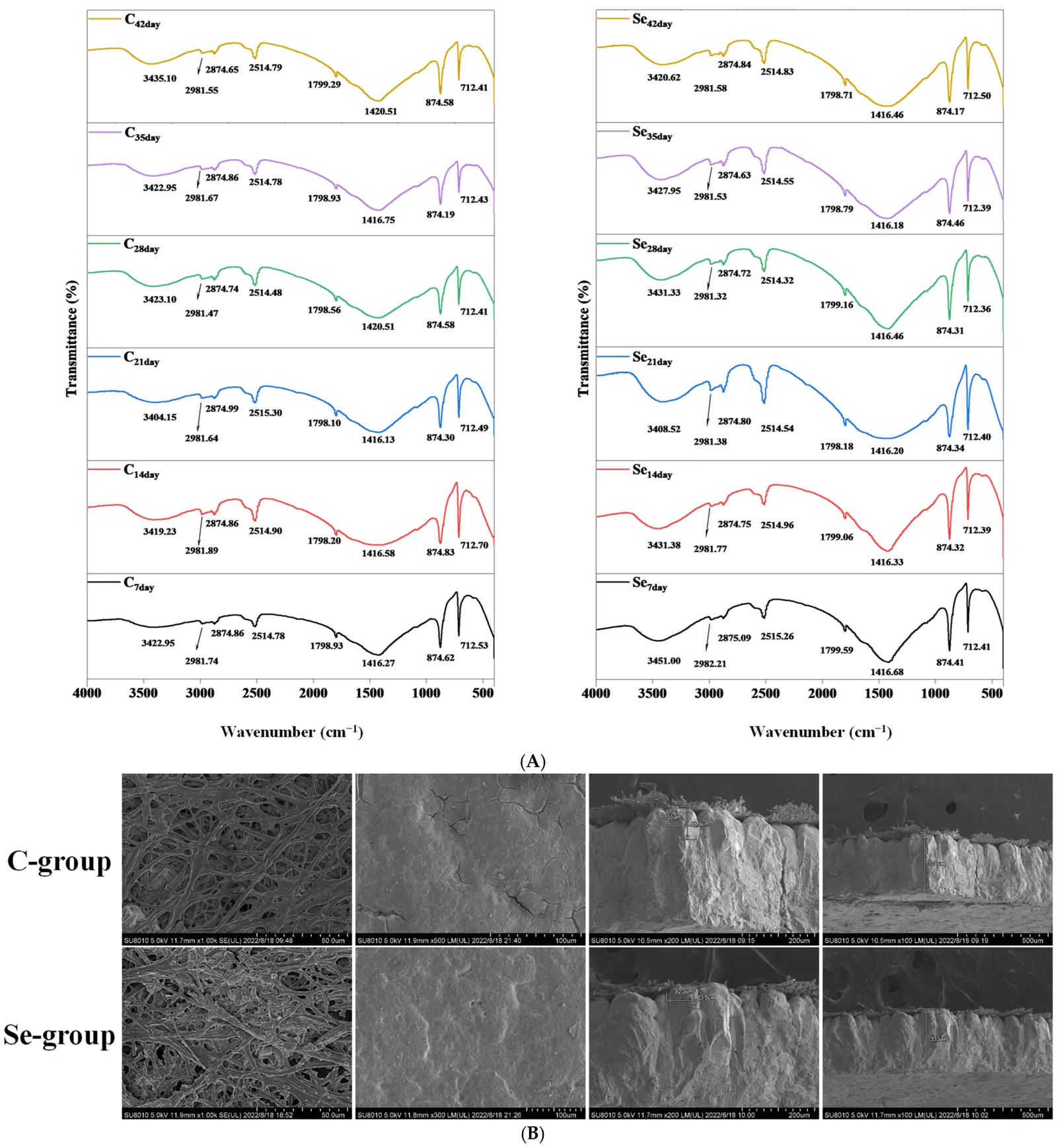
Changes in FTIR of eggshell (**A**) and microstructure of eggshells (**B**) during the 42-day SeMet supplementation period. Panel (**A**): Spectra were acquired via potassium bromide pellet compression over 4000–400 cm^−1^ with 4 cm^−1^ resolution and 32 scans using a spectrometer (Nicolet iS10, Waltham, MA, USA). Panel (**B**): Samples (~1 cm^2^) from the equatorial region of eggshells were boiled in 2% NaOH for 10 min, dried at room temperature, and observed under a Hitachi SEM (SU8010, Tokyo, Japan) at an accelerating voltage of 3.0 kV.

Figure 2A shows the FTIR spectroscopy results of the eggshells during the feeding period. The peaks in the range of 3700–3000 cm^−1^ are related to O-H stretching and N-H stretching vibrations; the peaks near 713, 872, and 1420 cm^−1^ are caused by CO_3_^2−^ asymmetric stretching vibration, out -of -plane bending vibration, and C-O antisymmetric stretching vibration, respectively [16]. The peak near 1420 cm^−1^ in the Se-group is greater than that in the C-group, indicating that there is more calcium carbonate in Se-group eggshells. The higher calcium carbonate content in Se-group eggshells relative to the C-group may be attributed to selenium promoting the activation of VD, improving the utilization and deposition of calcium in the body, and improving the quality of eggshells.

#### 3.3.3. Changes in Microstructure of Eggshells

Eggshell is a unique natural protective barrier that can protect eggs and embryos from microbial invasion and physical damage during embryonic development. Meanwhile, the eggshell also allows the exchange of water and air through the pores. The structural characteristics of the eggshell are important for its strength, such as the density of the nipple nodes, the effective thickness of the papillae layer, and the width of the papillae gap [18]. Figure 2B shows the ultrastructure of eggshells from hens fed for 42 days. Image analysis revealed that the average pore size in the Se group was approximately 5.61 μm, compared to 9.48 μm in the C group. The results showed that the cross-section of the Se-group eggshells was more compact and denser, and the width of the mastoid bodies was significantly lower than that of the C-group. Additionally, the thickness of the palisade layer of the Se-group eggshells was significantly higher than that of the C-group. Therefore, eggs produced by laying hens fed with SeMet have a smoother surface and a thicker and denser surface fiber diameter, endowing the eggshell with better mechanical properties, which can effectively protect the internal components of the egg from external environmental influences and reduce losses caused by eggshell breakage during transportation.

### 3.4. Analysis of Egg White

#### 3.4.1. Changes in Amino Acid Content of Egg White

As shown in Table 5, the egg white sample was detected to contain 17 total amino acids. Compared with the C-group, the amino acid content of the Se-group showed an overall upward trend, with a significant increase in the content of aspartic acid, serine, methionine, isoleucine, and proline (*p* < 0.05). On the 42nd day, the ratio of essential amino acids to total amino acids in Se-group egg white was 42.53%, indicating that the Se-group had an excellent egg white protein pattern.

**Figure 3:**
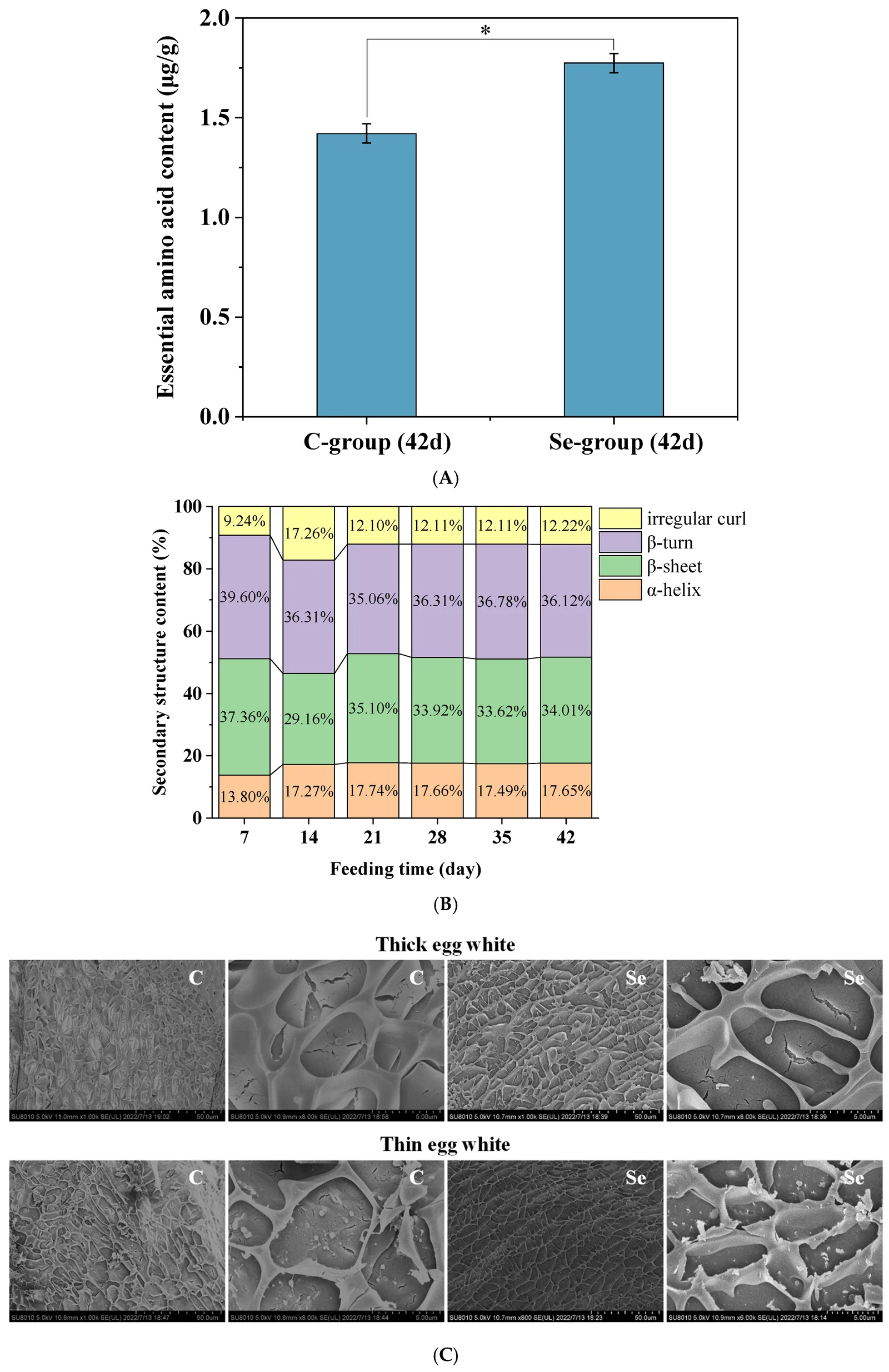
Changes in free essential amino acids (**A**), protein secondary structure (**B**), left: C-group; right: Se-group, and Cryo-SEM observation (**C**) of egg white during the 42-day SeMet supplementation period. Panel (**A**): Free essential amino acids were extracted with 5% trichloroacetic acid, incubated at 4 °C overnight, centrifuged at 12,000× *g* for 30 min at 4 °C, and filtered through a 0.2 μm membrane prior to analysis. Panel (**B**): Protein secondary structures were derived from ATR-FTIR spectra acquired under the same parameters as Panel (**A**). Panel (**C**): Samples were sublimated at −90 °C for 15 min, sputter-coated with gold, and imaged at −140 °C under 5 kV accelerating voltage and 10 mA beam current; scale bar = 5.0 μm. * represents the difference analysis between different treatment groups at the same feeding time (*p* < 0.05), and no annotation indicates no significant difference.

As the days of selenium enrichment in the diet increased, the content of methionine in free amino acids in egg white also gradually increased. As shown in Table 6, the egg white sample was detected to contain 12 amino acids. Figure 3A shows that compared with the C-group, the content of arginine, phenylalanine, tyrosine, leucine, isoleucine, and methionine in the Se-group gradually increased. Methionine is involved in methyl metabolism and the synthesis of other sulfur-containing amino acids, especially cysteine. The essential amino acid content of egg white in the Se-group on day 42 was higher than that of the C-group.

#### 3.4.2. Changes in FTIR of Egg White

Figure 3B shows the changes in the secondary structure of egg white protein across the experimental periods. The results show that β-sheets and β-turns in the egg whites of the Se-group and C-group accounted for over 65% of the secondary structure, indicating that these were the main secondary structures in egg white. As the feeding time continued to increase, the content of β-sheets and β-turns gradually decreased, while that of α-helices and irregular curls gradually increased. However, the difference between the secondary structure of egg white protein in the Se-group and C-group was not significant, which indicates that SeMet does not significantly affect the secondary structure of protein.

#### 3.4.3. Changes in Microstructure of Egg White

Figure 3C shows the microstructures of thick and thin egg whites in the C-group and Se-group under freeze-scanning electron microscopy. The C-group and Se-group showed a porous network structure in both thick and thin egg whites. However, the network structure of concentrated egg white had a large convex structure, and the network of thick egg white was more complete, while the image of thin egg white showed more small fragments at 5.0 μm.

### 3.5. Analysis of Egg Yolk

#### 3.5.1. Changes in Amino Acid Content of Egg Yolk

**Figure 4:**
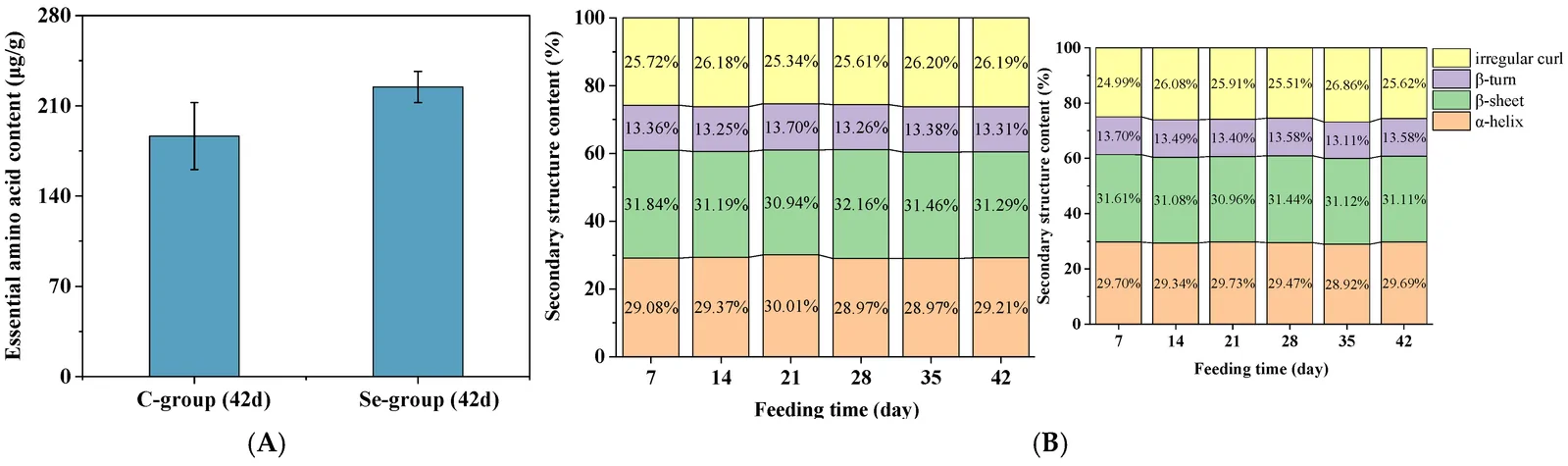
Changes in free essential amino acid content (**A**), and protein secondary structure (**B**) of egg yolk. during the 42-day SeMet supplementation period. Panel (**A**): Total amino acids were determined after acid hydrolysis with 6 mol/L HCl at 110 °C for 22 h, and free amino acids were extracted with 5% trichloroacetic acid; both were analyzed using a amino acid analyzer (Hitachi L-8900, Hitachi, Tokyo, Japan). Panel (**B**): Protein secondary structures were derived from ATR-FTIR spectra acquired under the same parameters as Figure 2A.

Table 7 shows that the egg yolk contains a total of 17 total amino acids. Compared to C-group, the amino acid content of the Se-group showed an overall upward trend, with a significant increase in the content of aspartic acid, threonine, serine, glutamic acid, glycine, and alanine. Table 8 shows that the egg yolk sample contains 18 free amino acids. With the increase in days receiving the selenium-enriched diet, the trend of amino acid changes was not significant (*p* > 0.05), but the amino acid content at 42 days showed an increasing trend compared to C-group, especially aspartic acid and cysteine. Figure 4A shows that the essential amino acid content in the egg yolk of the Se-group on the 42nd day is higher than that of the C-group.

#### 3.5.2. Changes in FTIR of Egg Yolk

Figure 4B shows that the secondary structure of egg yolk protein is mainly composed of α-helices and β-sheets (accounting for over 60% of the total), and the content of β-sheets is slightly higher than that of α-helices. In addition, the content of β-turns in egg yolk protein is lower than that of α-helices and β-sheets, and the proportion of irregular curl content is the lowest. The changes in the secondary structure content of egg yolk across the experimental periods were not significant in either the C-group or the Se-group, indicating that feeding time did not significantly affect the structure of egg yolk protein, while the content of β-sheets in the Se-group was slightly lower than that in the C-group.

#### 3.5.3. Changes in Fatty Acid Content of Egg Yolk

Analysis of yolk fatty acid profiles identified 17 fatty acids, including 8 saturated, 5 monounsaturated, and 4 polyunsaturated fatty acids (Table 9). SeMet supplementation increased the overall content of saturated and unsaturated fatty acids relative to the C-group. Most notably, n-3 polyunsaturated fatty acid (PUFA) content rose from 0.01 mg/g to 0.05 mg/g, while the n-6/n-3 PUFA ratio decreased from 2246.24 to 779.48, indicating a substantial improvement in the nutritional quality of yolk lipids.

**Figure 5:**
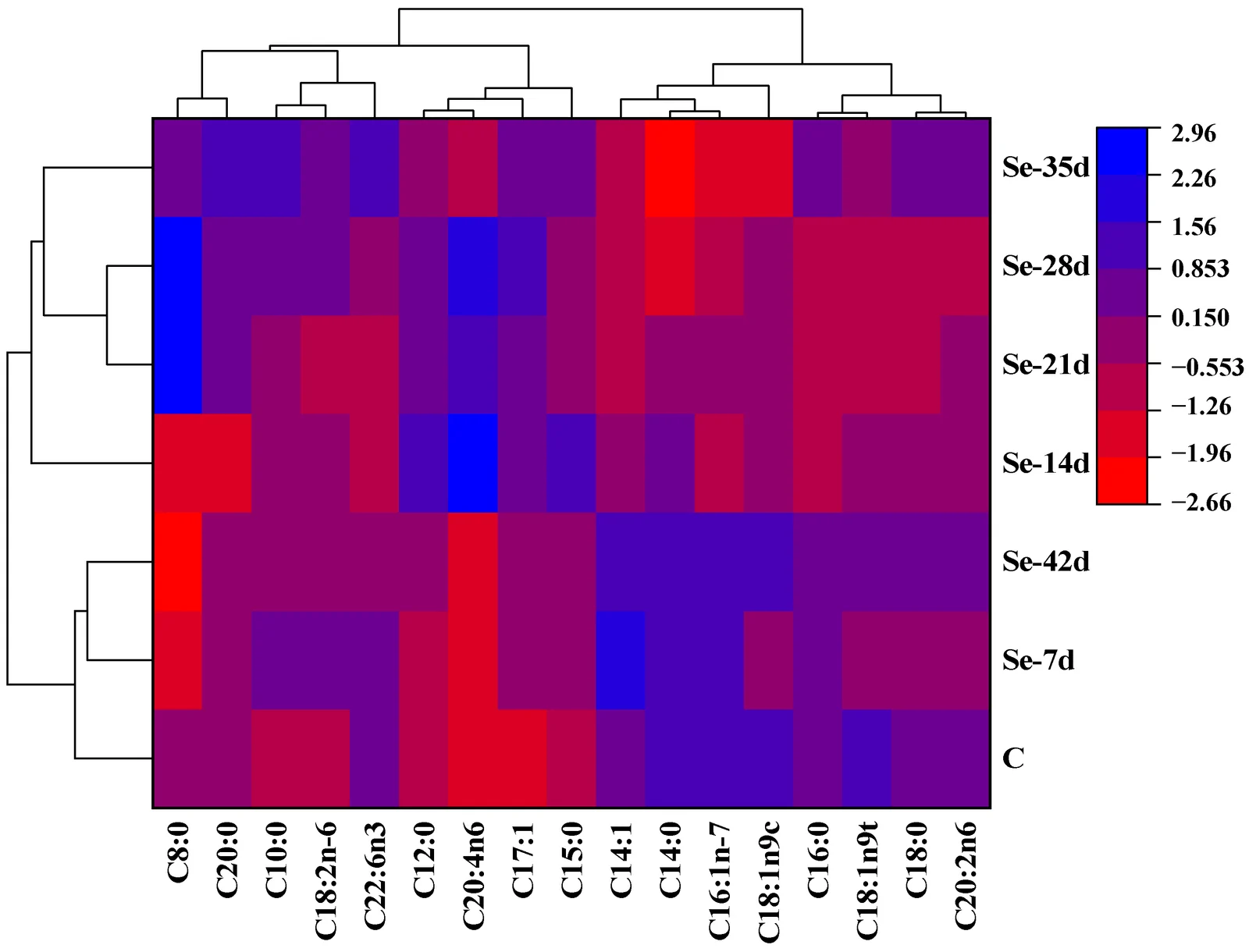
Changes in clustering patterns of fatty acids in egg yolk (hierarchical cluster analysis) during the 42-day SeMet supplementation period. Fatty acids were extracted with chloroform–methanol (2:1, *v*/*v*), methylated according to GB 5009.168-2016 (China, 2016), and analyzed by GC-MS (Agilent 8890-7000D, DB-5 MS column, Agilent Technologies, Santa Clara, CA, USA). Hierarchical cluster analysis was performed using Ward’s method with squared Euclidean distance as the similarity metric.

Systematic cluster analysis (hierarchical cluster analysis, HCA) was conducted on different days of selenium enrichment in the Se-group and the C-group. HCA was performed using the square of intergroup chains, and Euclidean distance was used to measure the homogeneity of fatty acids between Se-group egg yolk samples based on different feeding durations. As shown in Figure 5, all samples are divided into three clusters, indicating significant differences in fatty acids between egg yolk samples at different times under selenium-rich conditions. The first cluster is the C-group, Se-7 d, and Se-42 d, while the second cluster is for samples of Se-14 d, Se-21 d, Se-28 d, and Se-35 d. samples. The C-group, Se-7 d, and Se-42 d samples have higher C10:0, C14:0, C16:0, C16:1 nt, C18:1n9t, and C18:1n9c ratios, while the Se-14 d, Se-21 d, Se-28 d, and Se-35 d samples have higher C20:4n6 and C20:0 ratios.

#### 3.5.4. Lipidomics Analysis

**Figure 6:**
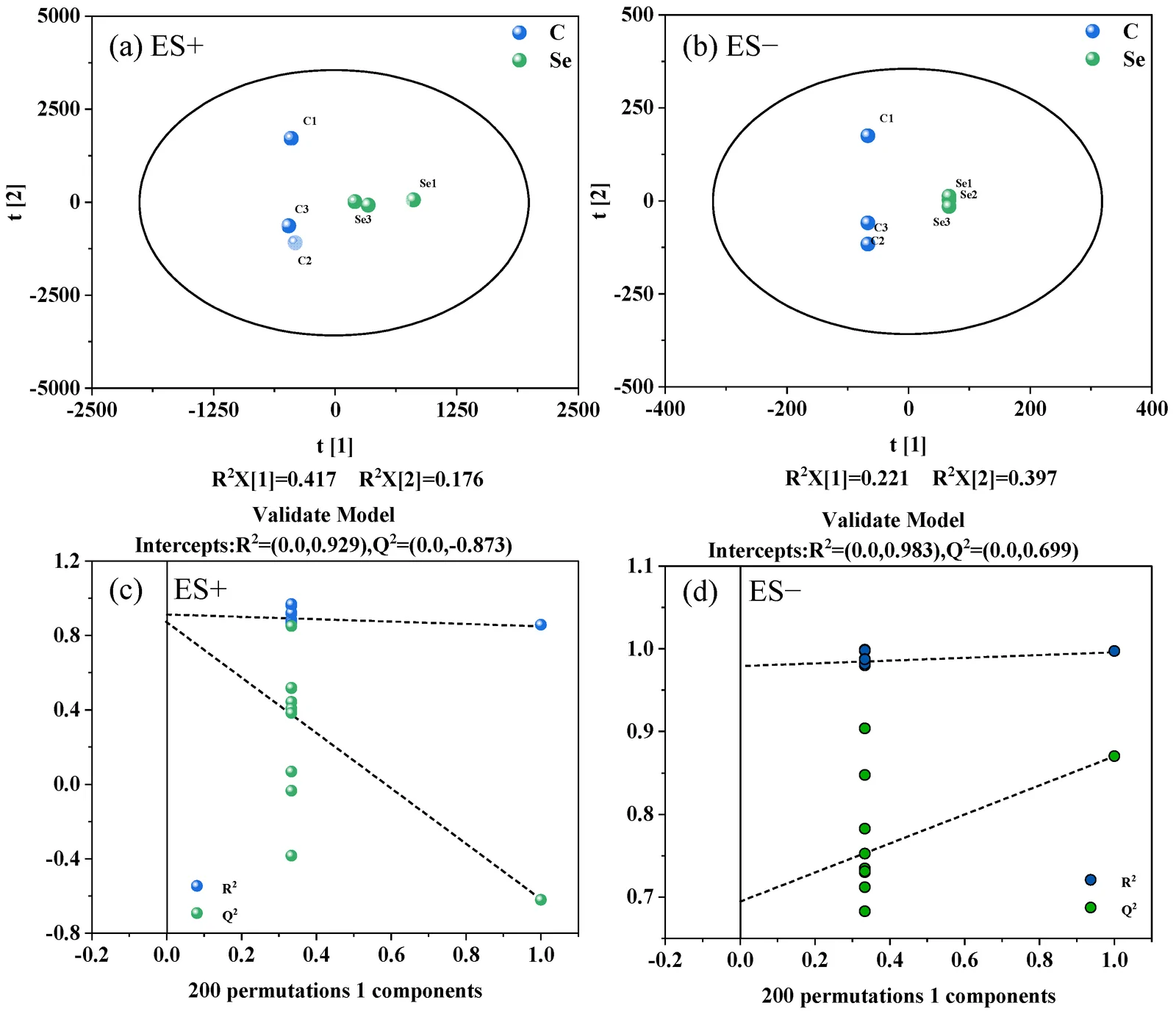
Changes in OPLS-DA model analysis of egg yolk during the 42-day SeMet supplementation period. (**a**) OPLS-DA score plot under ES+ mode; (**b**) OPLSDA score plot under ES− mode; (**c**) Permutation test plot (200 permutations) for model validation under ES+ mode; (**d**) Permutation test plot (200 permutations) for model validation under ES− mode.

To better isolate and understand the lipid changes between all groups, orthogonal partial least squares discriminant analysis (OPLS-DA) model analysis was performed on lipid metabolites. Figure 6 shows a clear separation trend between the C-group and Se-group, indicating significant differences in lipids between the two groups. Orthogonal partial least squares discriminant analysis (OPLS-DA) revealed clear separation between the C-group and Se-group, with model evaluation metrics (R2Y = 0.857 in ES+, 0.997 in ES−; Q2 = 0.870 in ES−) confirming reliability for identifying lipidomic differences.

**Figure 7:**
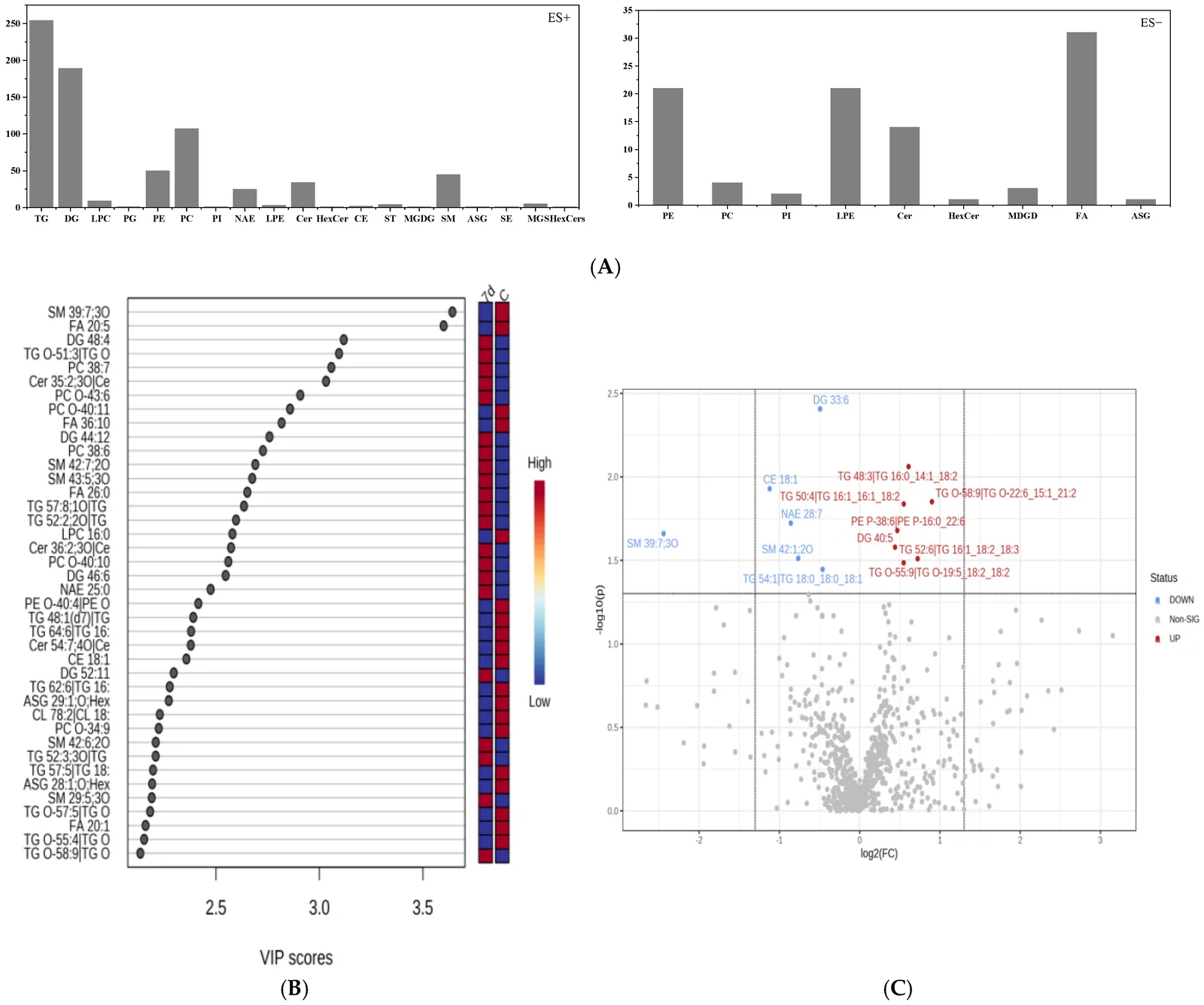
Lipid subclasses analysis (**A**), VIP map, and volcano map of egg yolk lipid (**B**) and clustering heat map of different lipids in egg yolk (**C**) during the 42-day SeMet supplementation period. Lipids were extracted with chloroform-methanol (2:1, *v*/*v*) and separated on an ACQUITY UPLC BEH C18 column (100 mm × 2.1 mm, 1.7 μm), followed by detection via AB SCIEX TRIPLETOF 5600 quadrupole time-of-flight tandem mass spectrometry in positive and negative ion modes. Differential lipids were screened by VIP > 1.2 and *p* < 0.05. The heatmap displays results from three biological replicates per group, with tighter intra-group clustering and distinct inter-group separation.

Figure 7A shows that 735 lipid subclasses were isolated and identified in ES+ and 98 in ES−. Figure 7B shows 40 lipid classes with VIP values ranging from high to low in egg yolks from the C-group and Se-group based on ES+ and ES− modes. According to FC > 1.2 and *p* < 0.05, a total of 13 differential lipid compounds were identified (FC > 1.2, *p* < 0.05), comprising 7 upregulated and 6 downregulated species. Notably, triglycerides (TG, six species) were consistently upregulated, while diglycerides (DG, two species) were downregulated (Figure 7A,B), indicating SeMet suppresses lipid hydrolysis in yolk.

Cluster heatmap analysis was employed to explore the similarity of identified lipid molecules in the C-group and Se-group. In Figure 7C, the lipid clustering between the three replicates within the group is similar, while the inter- group clustering is greater than the intra-group clustering, and the module colors representing up- and down-regulation are opposite. This clustering effect is better, indicating significant differences in lipid composition and function between the C-group and Se-group.

#### 3.5.5. Changes in Microstructure of Egg Yolk

**Figure 8:**
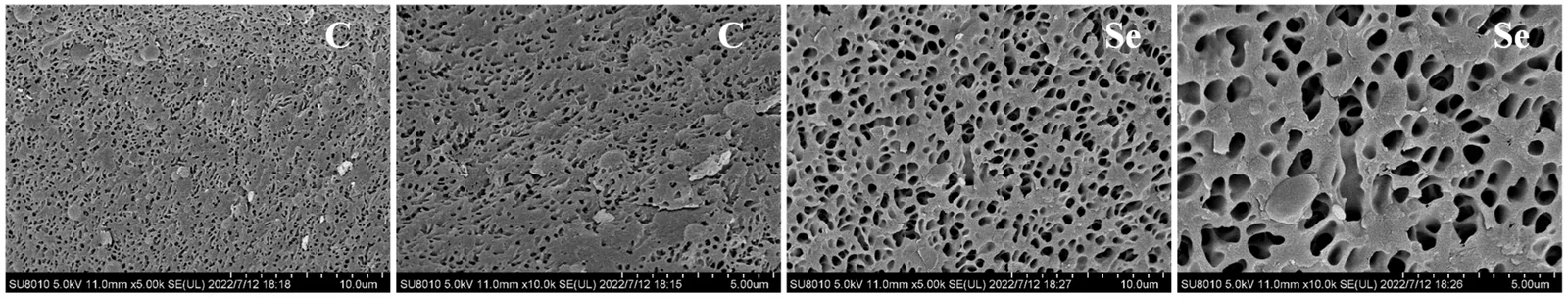
Changes in microstructural features of egg yolk (Ccryo-SEM porous honeycomb structure) during the 42-day SeMet supplementation period. Samples were sublimated at −90 °C for 15 min, sputter-coated with gold, and imaged at −140 °C under 5 kV accelerating voltage and 10 mA beam current. The average pore size of the C-group yolk was ~0.31 μm, while that of the Se-group was ~2.93 μm.

The microstructure of Se-group and C-group egg yolks was determined using cryoelectron microscopy (Hitachi, Tokyo, Japan), and the results are shown in Figure 8. The Cryo-SEM image of the egg yolk presents a porous honeycomb shape. Image analysis indicated that the average pore size of the yolk microstructure in the C-group was approximately 0.31 μm, whereas that in the Se-group was approximately nine times larger, at 2.93 μm. The honeycomb-like structure of the C-group egg yolk is significantly denser than that of the Se-group. Meanwhile, spherical objects embedded in the pores may reflect differences in lipid–protein organization [19].

## 4. Discussion

Different forms of selenium products have been extensively studied, including SeY, SeS, SeMet, and SeN. Besides these organic selenium sources, inorganic selenium sources such as sodium selenite and sodium selenate are still widely used in commercial poultry production owing to their low cost. Nevertheless, issues including low intestinal absorption efficiency, environmental selenium emissions, and potential toxicity limit their large-scale application. The development and utilization of diverse selenium sources have attracted extensive attention within livestock nutrition. Although many studies have attempted to develop novel organic selenium preparations, few have systematically compared how various selenium types synchronously regulate multidimensional egg quality. The present study fills this research gap by comprehensively characterizing the physicochemical and structural traits of eggs rather than merely focusing on laying performance and selenium deposition. To elucidate the underlying mechanisms beyond conventional production metrics, this study characterizes concurrent changes in mineral composition, amino acid profiles, structural characteristics, and lipid components to systematically clarify how methionine selenium (SeMet) supplementation enhances egg quality.

Some researchers have shown that selenium supplementation has no effect on laying rate [20]. However, Han et al. [11] reported that its combination with SS or SY supplementation can increase egg production. Organic selenium is superior to inorganic selenium in improving production performance, and selenium-enriched yeast has more effectively improved production performance than sodium selenate [21,22]. Wu et al. studied the effects of different sources and levels of SeMet on the production performance of breeding chickens, reporting significant improvements in egg laying and hatching rates [23]. In addition, Zai et al. [24] reported that supplementing yeast selenium increased the egg weight of Assel chickens. After adding SeMet to this experiment, the average egg weight was increased, which is consistent with the experimental results of Zai. Therefore, SeMet effectively improved the production performance of laying hens.

The Haugh unit is an important indicator reflecting the freshness of eggs, indicating the degree of thinning of the thick protein in poultry eggs. As the freshness of eggs decreases, the concentration of concentrated protein decreases, resulting in a decrease in the protein functional properties and a decrease in the Haugh unit [25]. Egg quality deterioration during storage is mainly driven by protein oxidation, water migration, and lipid peroxidation. Oxidative modification of thick egg white proteins will destroy their spatial structure, weaken the gel characteristics, and finally reduce the Haugh unit. As an essential component of antioxidant enzymes, selenium can mitigate protein carbonylation and inhibit the degradation of ovomucin, which is the core protein maintaining thick egg white viscosity. The improved egg freshness indicators observed in the SeMet group imply that dietary SeMet may activate the antioxidant defense system in laying hens and further transfer antioxidant activity to eggs, delaying the quality decay of eggs during shelf storage. The height of the air chamber can also reflect the freshness of the egg, where the lower the height of the air chamber, the greater the freshness of the egg. In addition, the yolk index can be used to measure the freshness of egg yolks, where the higher the yolk index, the fresher the egg yolk [26]. In this study, SeMet showed a positive impact on egg quality at different feeding periods. Payne et al. found that the Haugh unit and egg yolk index of organic selenium supplemented with 0.15, 0.30, 0.60, and 3.00 mg/kg in the diet were higher than those in the basal diet group [27]. Zia et al. reported that adding selenium yeast can increase the egg yolk index and Haugh unit [22]. These phenotypic improvements correspond to the observed increase in essential amino acid content (Table 5 and Table 7) and the maintenance of native protein secondary structures (Figure 3B and Figure 4B), suggesting that SeMet preserves protein functionality while enhancing nutritional composition.

Eggshell quality is a critical concern in laying-hen production, and thicker eggshells are more conducive to successful egg transportation. Eggshell breakage remains one of the major economic losses in the laying hen industry, especially under intensive breeding, high temperature, and other stress conditions. Heat stress and oxidative stress will interfere with calcium transport in the shell gland and disrupt mammillary layer formation. Existing evidence confirms that selenium can alleviate oxidative damage to shell gland epithelial cells [28]. In this study, the optimized shell micromorphology and elevated calcium and phosphorus deposition indicated that SeMet could relieve oxidative stress of shell gland tissues, thereby creating favorable conditions for biomineralization of eggshells. In practical production, improved shell strength can effectively reduce breakage-related losses during sorting, transportation, and sales, which enhances the economic benefits of laying hen farms. Poor eggshell strength can lead to an increase in egg breakage rate, which in turn affects economic benefits. Hocking et al. reported a correlation between eggshell strength and eggshell thickness, with eggshell thickness being the main factor affecting eggshell strength. Baylan et al. reported that yeast selenium significantly improved eggshell weight and thickness compared with sodium selenite [29]. The strength of eggshells is related to the plasma mineral content of laying hens. In the early stage of shell formation, supplementing trace minerals can promote early fusion and improve the mechanical strength of eggs [30]. The increase in eggshell strength (+29.24%) and weight (+0.81%) was directly associated with the elevated levels of Ca and P in the Se-group eggshells (Figure 1A), as well as the FTIR result showing increased calcium carbonate content (Figure 2A). Adding Zn-Met to the diet can promote zinc deposition in the liver and eggshell gland of laying hens in the later stages of laying, thereby increasing the activity of carbonic anhydrase in the liver and eggshell gland and promoting calcium deposition in the eggshell, leading to an increase in thickness and strength and ultimately reducing the breaking rate [31]. The addition of 40 mg/kg MHA-Zn to dietary supplements can improve eggshell quality by promoting Ca deposition and carbonic anhydrase activity [32]. Previous studies have shown that trace minerals (e.g., Zn) improve eggshell quality by promoting Ca deposition partly through modulating carbonic anhydrase activity, and a similar mechanism may contribute to the enhanced shell quality observed here. In addition, the color of the eggshell can be used as an indicator to measure the strength of the eggshell. The darker the color of the eggshell of the same variety, the greater the strength of the eggshell [33]. The deposition of biliverdin on the eggshell can affect the color of the eggshell, and protoporphyrin IX is also considered the main factor affecting the color of brown eggshells. This research found that, compared with the C-group, the Se-group had a darker color and increased eggshell strength. Adding Zn-Met to the diet can promote zinc deposition in the eggshell, thereby increasing the activity of carbonic anhydrase. Hence, the results show that SeMet effectively improves the quality of eggshells and reduces unnecessary economic losses. This strengthening is further supported by the Cryo-SEM observation of a denser palisade layer and a reduced mammillary knob width in Se-group eggshells (Figure 2B), thereby directly linking mineral deposition and microstructural reorganization to mechanical performance.

In recent years, with the deepening of research on the biological functions of selenium, eggs have become the most ideal carrier for producing organic selenium-rich functional foods. Selenium deficiency is prevalent in many regions worldwide, and dietary intervention via selenium-enriched animal-derived food is regarded as a safer supplementation strategy compared with direct selenium pills. Different chemical forms of selenium in food exhibit distinct absorption efficiency in the human intestinal tract. Unlike inorganic selenium, SeMet can be stored in body protein pools and sustain selenium supply for humans over a longer period. Most previous studies only determined total selenium concentration in eggs; this work further analyzed free amino acid profiles and protein secondary structure, which provided more comprehensive data for evaluating the nutritional value of selenium-enriched eggs as a functional food. The results showed that the selenium content in eggs could change in a short period of time with the addition of selenium supplementation to the diet. After 14 days of feeding, selenium levels in eggs from the Se-group were significantly higher than those from the C-group. Methionine is transported via specific amino-acid transporters located in the intestinal mucosa. After absorption, SeMet is easily incorporated into tissue proteins in a nonspecific and unregulated manner [34]. The nutritional value of proteins mainly depends on their amino acid composition. Dietary supplements can affect the content of protein and amino acids in eggs [35]. In addition, compared to inorganic selenium, selenium present in the form of SeMet has the highest bioavailability; therefore, foods with high SeMet content have greater bioavailability. The composition of free amino acids is an important indicator for metabolic research in biotechnology and biomedicine, and it is also a frequently tested parameter in food nutritional analysis. During the gastrointestinal digestion of food, proteins are hydrolyzed into various peptides and free amino acids. These protein hydrolysates, along with other nutrients, can participate in hormone regulation in the body [36]. The results showed that egg yolk contains 18 free amino acids, and egg white contains 12 free amino acids. Free Met displayed an overall upward trend across experimental periods. This finding suggests that selenium-rich egg yolk may release more Met upon human gastrointestinal digestion, which is conducive to human digestion and absorption. In general, feeding SeMet is beneficial for improving egg production performance and maintaining the sustainability of egg production in laying hens. The concurrent increase in total amino acids (Table 5 and Table 7) and free methionine (Table 6 and Table 8) confirms that SeMet serves as both a mineral supplement and a substrate for sulfur-containing amino acid enrichment, thereby elevating the overall nutritional profile of the egg.

The lipids of egg yolk mainly exist in the form of lipoproteins, consisting of 62–65% triglycerides, 30–33% phospholipids, and 4–5% cholesterol. In the present study, dietary selenium supplementation increased the overall contents of both saturated and unsaturated fatty acids. This finding is consistent with the report by Gürbüz et al., which may be attributed to the interaction between selenium and PUFAs [37]. Polyunsaturated fatty acids such as n-3 PUFAs are susceptible to oxidative degradation, which leads to off-flavor generation and shortened shelf life of eggs and egg products. Stabilization of the lipid matrix by SeMet not only retains the nutritional value of yolk lipids but also helps maintain sensory quality during processing, such as liquid egg products, preserved eggs, and baked foods. The inhibitory effect of SeMet on lipid hydrolysis and peroxidation reveals its dual value: simultaneously improving egg nutrition and extending the storage stability of egg products. Egg yolk lipids are known to have significant nutritional importance, particularly in the development of brain function. Further investigation into the lipid composition of egg yolks is necessary. Consuming egg yolks could increase the availability of anti-inflammatory lipid moieties and structural lipids. Previous research has mostly focused on the cholesterol content of eggs and the potential harmful consequences of this lipid on health, while neglecting the important role of cholesterol in membranes and its properties as a precursor to other essential steroids. Currently, strategies to reduce egg cholesterol levels are being evaluated, and our experimental results indicate that SeMet effectively reduces cholesterol levels.

TGs are the main energy storage pool, and egg yolk contains a large amount of asymmetric triacylglycerol (TAG), which is a storage compound and the main lipid resource available for embryos. Egg yolk also contains polar lipids (PLs) that form membrane structural components and sterols (STs), which are involved in hormone and vitamin synthesis [38]. The main component of natural oils or fats, TG, is produced through lipid hydrolysis and is gradually converted into DG, monoglycerides (MGs), and glycerol through hydrolysis, accompanied by the release of fatty acids [39]. Therefore, these results suggest that SeMet may alleviate lipid hydrolysis. The upregulation of glycerol phospholipids, such as LPC, is due to phospholipid degradation or enzymatic hydrolysis, while glycerol phospholipids (GPs), such as PE and phosphatidylcholine (PC), are downregulated. The upregulation of DG is closely related to the degradation and oxidation of TG [40]. The results showed that, compared to the C-group, TG in the Se-group showed an upward trend, while DG showed a downward trend, correlating with the possibility that SeMet may inhibit chain breakage, side-chain modification, or fat decomposition, thereby preventing lipid peroxidation. The lipidomic shifts—specifically the upregulation of triglycerides and downregulation of diglycerides (Figure 7)—correlate with the observed increase in n-3 PUFA content (Table 9), indicating that SeMet stabilizes the yolk lipid matrix by suppressing hydrolytic breakdown. The lipidomic shifts, particularly the upward trend in triglycerides and reduced diglycerides, parallel the increased n-3 PUFA content observed in Se-group yolks (Table 9). Given that n-3 PUFAs are typically esterified at the sn-2 position of triglycerides, the accumulation of TAG likely protects these labile fatty acids from oxidative loss. This metabolic shift appears to coincide with the significantly reduced air chamber height (−21.51%, Table 3), suggesting a potential role in mitigating moisture loss during storage. Notably, these lipid alterations occur alongside a denser eggshell microstructure (Figure 2B) and elevated essential amino acid profiles (Table 5, Table 6, Table 7 and Table 8). While these concurrent modifications suggest a synergistic improvement in egg quality, further validation is required to confirm whether lipid remodeling directly influences shell integrity or nutrient retention.

Compared with ordinary eggs, the egg white of selenium-rich eggs has a clear, thin, and tight egg liquid, a thick egg white, and toughness, indicating that SeMet can, to some extent, reduce the loss of free water in the egg white. The main protein in egg white is egg white protein. Although selenium-rich eggs can change the amino acid composition in egg white, they have little effect on its secondary structure. This suggests that the SeMet selected in this study can enrich the nutrients of eggs without affecting other physicochemical properties of egg white. The preservation of native protein secondary structures despite compositional changes underscores that SeMet enhances the nutritional value without compromising the fundamental physicochemical properties of egg components.

Nevertheless, several limitations should be acknowledged in the current study. We only focused on the phenotypic changes in eggs after SeMet supplementation, and the molecular signaling pathways mediating shell gland biomineralization, ovarian lipid metabolism, and intestinal nutrient absorption require further verification at the gene and protein levels. Future research should explore the optimal supplemental dose of SeMet under different feeding environments and investigate the dynamic changes in the physicochemical properties of selenium-enriched eggs during long-term storage. Moreover, sensory evaluation and human bioavailability trials are necessary to support the industrial promotion of SeMet-derived selenium-enriched eggs.

## 5. Conclusions

According to this study, the dietary supplementation of SeMet improved the production performance and egg quality of Hy-Line Brown laying hens. Relative to the C-group, SeMet supplementation increased the laying rate by 5.42% and reduced the egg breakage rate by 66.67%. Meanwhile, SeMet treatment decreased the air chamber height, improved eggshell strength by 29.24% and shell pigmentation intensity by 31.26%, and promoted mineral deposition in the eggshell, egg white, and egg yolk. Nutritionally, SeMet elevated essential amino acid levels in both egg white and egg yolk and increased saturated and unsaturated fatty acid contents in egg yolk, while maintaining the native secondary structure of egg proteins. These findings fill existing research gaps by systematically characterizing the multidimensional quality traits of different egg compartments. Combined with the high bioavailability of SeMet, the optimized n-6/n-3 PUFAs profile and inhibited yolk lipid hydrolysis observed herein provide reliable support for the development of functional selenium-enriched eggs. These findings also provide a feasible theoretical basis and technical guidance for optimizing laying hen nutritional regimens and improving egg storage and processing strategies.

## Figures and Tables

**Table 1:** Premix composition and nutrient content.

Composition	Content (%)
Crude protein	30
Crude fiber	9
Crude ash	38
Calcium	6–12
Phosphorus	0.6
Sodium chloride	0.3–2
Methionine	0.6

**Table 2:** Conditions of positive and negative ions in mass spectrum.

Parameter	Positive-Ion		Negative-Ion	
	ESI-MS	ESI-MS2	ESI-MS	ESI-MS2
Scan Type	TOF	TOF	TOF	TOF
Ion Spray Voltage Floating	5500 V	5500 V	−4500 V	−4500 V
Source Gas 1	60 psi	60 psi	60 psi	60 psi
Source Gas 2	60 psi	60 psi	60 psi	60 psi
Curtain Gas	35 psi	35 psi	35 psi	35 psi
Temperature	550 °C	550 °C	550 °C	550 °C
Declustering Potential	80 V	80 V	80 V	80 V
Collision Energy	10 V	40 V	−10 V	−40 V
Collision Energy Spread		20 V		20 V

**Table 3:** Changes in egg freshness indicators (air chamber height, protein height, Haugh units, and egg yolk index) during the 42-day SeMet supplementation period.

Feeding Time (Day)	Air Chamber Height		Protein Height		Haugh Units		Egg Yolk Index	
	C	Se	C	Se	C	Se	C	Se
7	3.77 ± 0 18 ^a^	3.77 ± 0.07 ^a^	12.85 ± 0.72 ^a^	12.49 ± 0.74 ^a^	109.09 ± 2.29 ^c^	109.14 ± 3.27 ^a^	0.38 ± 0.03 ^a^	0.39 ± 0.01 ^a^
14	3.20 ± 0.17 ^ab^	3.13 ± 0.26 ^b^	11.64 ± 0.44 ^a^	11.74 ± 0.17 ^b^	109.03 ± 2.90 ^c^	109.73 ± 1.80 ^a^	0.37 ± 0.06 ^a^	0.39 ± 0.06 ^a^
21	3.45 ± 0.34 ^a^	2.83 ± 0.15 ^b^	12.07 ± 0.30 ^a^	12.37 ± 0.18 ^b^	105.51 ± 0.97 ^bc^	109.61 ± 2.62 ^a^	0.37 ± 0.06 ^a^	0.39 ± 0.03 ^a^
28	3.80 ± 0.15 ^a^	2.73 ± 0.09 ^bc^	11.09 ± 1.53 ^a^	12.57 ± 0.22 ^b^	102.04 ± 3.40 ^ab^	109.66 ± 1.98 ^a^	0.37 ± 0.02 ^a^	0.39 ± 0.08 ^a^
35	2.56 ± 0.18 ^b^	2.13 ± 0.32 ^cd^	11.67 ± 0.20 ^a^	12.62 ± 0.19 ^b^	100.76 ± 2.13 ^a^	109.67 ± 4.06 ^a^	0.37 ± 0.01 ^a^	0.39 ± 0.00 ^a^
42	2.51 ± 0.23 ^b^	1.97 ± 0.20 ^d^	13.21 ± 1.65 ^a^	13.75 ± 1.51 ^c^	99.70 ± 6.34 ^a^	109.92 ± 4.75 ^a^	0.37 ± 0.00 ^a^	0.39 ± 0.01 ^a^

The values in the table represent the average value of three measurements ± the standard deviation. Different lowercase letters in the same row indicate significant differences (*p* < 0.05).

**Table 4:** Changes in mechanical properties of eggshell (thickness, shape index, strength, weight, and color) during the 42-day SeMet supplementation period.

Feeding Time (Day)	Thickness (mm)		Shape Index		Strength (N)		Weight (g)		Color	
	C	Se	C	Se	C	Se	C	Se	C	Se
7	0.41 ± 0.02 ^a^	0.42 ± 0.01 ^a^	1.27 ± 0.02 ^ab^	1.26 ± 0.01 ^ab^	36.55 ± 2.99 ^a^	47.40 ± 0.77 ^a^	7.68 ± 0.20 ^a^	7.30 ± 0.19 ^a^	22.83 ± 0.02 ^d^	23.07 ± 0.07 ^d^
14	0.42 ± 0.02 ^a^	0.42 ± 0.01 ^a^	1.26 ± 0.02 ^ab^	1.31 ± 0.01 ^a^	37.07 ± 2.86 ^a^	47.46 ±1.82 ^a^	7.48 ± 0.26 ^a^	7.56 ± 0.44 ^a^	25.93 ± 0.10 ^b^	23.54 ± 0.02 ^d^
21	0.40 ± 0.01 ^a^	0.44± 0.01 ^a^	1.22 ± 0.01 ^b^	1.27 ± 0.02 ^ab^	36.53 ± 2.79 ^a^	47.01 ± 4.55 ^a^	7.74 ± 0.29 ^a^	7.48 ± 0.53 ^a^	21.04 ± 0.02 ^e^	30.45 ± 2.30 ^c^
28	0.40 ± 0.03 ^a^	0.44± 0.01 ^a^	1.27 ± 0.01 ^ab^	1.29 ± 0.03 ^ab^	35.47 ±5.21 ^a^	47.67 ± 2.87 ^a^	7.24 ± 0.34 ^a^	7.90 ± 0.18 ^a^	31.44 ± 0.08 ^a^	32.34 ± 1.24 ^b^
35	0.39 ± 0.02 ^a^	0.41 ± 0.02 ^a^	1.22 ± 0.02 ^b^	1.21 ± 0.03 ^ab^	36.31 ±4.74 ^a^	47.69 ± 1.28 ^a^	7.28 ± 0.36 ^a^	7.28 ± 0.30 ^a^	23.32 ± 0.15 ^d^	33.24 ± 0.11 ^a^
42	0.42 ± 0.01 ^a^	0.42 ± 0.02 ^a^	1.30 ± 0.01 ^a^	1.28± 0.01 ^b^	36.94 ± 4.18 ^a^	47.73 ± 1.58 ^a^	7.38 ± 0.06 ^a^	7.44 ± 0.22 ^a^	24.92 ± 5.57 ^c^	32.71 ± 1.27 ^ab^

The values in the table represent the average value of three measurements ± the standard deviation. Different lowercase letters in the same row indicate significant differences (*p* < 0.05).

**Table 5:** Changes in total amino acid profiles (mg/g) of egg white during the 42-day SeMet supplementation period.

	C-7 d	Se-7 d	C-14 d	Se-14 d	C-21 d	Se-21 d	C-28 d	Se-28 d	C-35 d	Se-35 d	C-42 d	Se-42 d
Asp	79.98 ± 15.95 ^ab^	75.92 ± 11.02 ^b^	79.75 ± 0.37 ^ab^	101.82 ± 3.65 ^c^	80.54 ± 0.17 ^ab^	92.82 ± 9.20 ^bc^	80.20 ± 0.35 ^ab^	102.73 ± 10.11 ^c^	80.60 ± 0.39 ^ab^	102.99 ± 2.76 ^c^	80.59 ± 0.12 ^ab^	105.13 ± 0.89 ^c^
Thr	36.79 ± 4.77 ^a^	36.47 ± 3.68 ^a^	40.48 ± 0.61 ^a^	44.37 ± 1.69 ^b^	41.53 ± 0.33 ^a^	40.93 ± 3.55 ^ab^	41.10 ± 0.42 ^a^	44.95 ± 3.91 ^b^	41.85 ± 0.66 ^a^	44.85 ± 1.19 ^b^	41.75 ± 0.08 ^a^	45.80 ± 0.35 ^b^
Ser	56.99 ± 7.91 ^ab^	54.80 ± 6.05 ^a^	63.98 ± 1.37 ^ab^	65.31 ± 3.27 ^bc^	66.78 ± 0.67 ^ab^	60.99 ± 4.20 ^abc^	65.93 ± 0.82 ^ab^	68.50 ± 6.56 ^c^	67.41 ± 1.29 ^ab^	67.50 ± 0.77 ^c^	67.29 ± 0.17 ^ab^	69.08 ± 1.39 ^c^
Glu	107.65 ± 17.85 ^a^	101.87 ± 13.73 ^a^	105.30 ± 0.23 ^a^	129.89 ± 6.72 ^b^	105.75 ± 0.12 ^a^	121.34 ± 8.21 ^ab^	105.57 ± 0.21 ^a^	136.17 ± 12.89 ^b^	105.84 ± 0.24 ^a^	134.94 ± 1.74 ^b^	105.79 ± 0.10 ^a^	137.33 ± 3.10 ^b^
Gly	30.73 ± 2.67 ^a^	30.20 ± 2.28 ^a^	35.19 ± 0.44 ^a^	34.70 ± 1.17 ^b^	35.97 ± 0.22 ^a^	32.88 ± 2.13 ^ab^	35.75 ± 0.28 ^a^	35.61 ± 2.37 ^b^	36.24 ± 0.38 ^a^	35.08 ± 1.01 ^b^	36.15 ± 0.09 ^a^	35.89 ± 0.25 ^b^
Ala	53.83 ± 4.96 ^ab^	51.88 ± 4.37 ^a^	53.95 ± 0.06 ^ab^	59.72 ± 2.18 ^bc^	54.50 ± 0.14 ^ab^	57.48 ± 2.83 ^abc^	54.08 ± 0.51 ^ab^	62.39 ± 3.81 ^c^	54.09 ± 0.20 ^ab^	61.52 ± 0.91 ^c^	54.38 ± 0.31 ^ab^	62.94 ± 0.66 ^c^
Cys	22.28 ± 2.14 ^a^	21.86 ± 0.56 ^a^	29.10 ± 0.39 ^a^	27.35 ± 1.41 ^a^	30.21 ± 0.08 ^a^	23.16 ± 5.12 ^a^	29.73 ± 0.61 ^a^	23.32 ± 1.97 ^a^	30.16 ± 0.47 ^a^	26.15 ± 3.05 ^a^	30.20 ± 0.35 ^a^	25.82 ± 3.63 ^a^
Val	59.80 ± 6.21 ^ab^	56.17 ± 3.90 ^a^	62.32 ± 0.40 ^ab^	67.29 ± 0.84 ^bc^	63.36 ± 0.06 ^ab^	62.54 ± 6.10 ^abc^	62.92 ± 0.50 ^ab^	66.80 ± 4.75 ^bc^	63.30 ± 0.45 ^ab^	67.16 ± 3.54 ^bc^	63.41 ± 0.17 ^ab^	68.40 ± 2.49 ^bc^
Met	35.04 ± 6.71 ^ab^	31.48 ± 3.95 ^a^	37.16 ± 0.39 ^ab^	41.90 ± 0.55 ^bc^	38.11 ± 0.09 ^ab^	37.52 ± 4.96 ^bc^	37.71 ± 0.46 ^ab^	41.31 ± 4.52 ^bc^	38.16 ± 0.47 ^ab^	42.37 ± 1.69 ^bc^	38.16 ± 0.19 ^ab^	43.07 ± 1.14 ^c^
Ile	43.97 ± 5.85 ^ab^	40.62 ± 3.94 ^a^	49.92 ± 0.71 ^ab^	50.17 ± 0.94 ^bc^	51.58 ± 0.12 ^ab^	46.19 ± 4.63 ^abc^	50.83 ± 0.90 ^ab^	50.69 ± 4.70 ^bc^	51.60 ± 0.85 ^ab^	50.64 ± 1.91 ^bc^	51.67 ± 0.38 ^ab^	51.66 ± 1.01 ^c^
Leu	78.69 ± 9.75 ^a^	74.69 ± 7.11 ^a^	78.30 ± 0.03 ^a^	91.27 ± 1.24 ^b^	78.24 ± 0.04 ^a^	83.76 ± 9.02 ^ab^	78.41 ± 0.05 ^a^	91.52 ± 8.50 ^b^	78.46 ± 0.10 ^a^	92.19 ± 3.76 ^b^	78.26 ± 0.18 ^a^	94.22 ± 1.92 ^b^
Tyr	36.44 ± 4.22 ^a^	36.03 ± 0.30 ^a^	38.72 ± 0.16 ^a^	35.43 ± 0.43 ^a^	39.03 ± 0.07 ^a^	37.10 ± 0.55 ^a^	39.09 ± 0.13 ^a^	37.61 ± 0.99 ^a^	39.30 ± 0.10 ^a^	35.60 ± 1.78 ^a^	39.08 ± 0.25 ^a^	36.37 ± 1.09 ^a^
Phe	56.42 ± 7.19 ^a^	53.54 ± 1.60 ^a^	54.62 ± 0.33 ^a^	56.56 ± 0.73 ^a^	55.42 ± 0.11 ^a^	57.16 ± 1.31 ^a^	55.09 ± 0.45 ^a^	59.34 ± 0.95 ^a^	55.48 ± 0.37 ^a^	57.05 ± 2.52 ^a^	55.38 ± 0.33 ^a^	58.18 ± 1.54 ^a^
Lys	56.17 ± 5.16 ^a^	55.36 ± 3.32 ^a^	54.64 ± 0.06 ^a^	63.42 ± 1.06 ^b^	54.37 ± 0.04 ^a^	60.13 ± 4.47 ^ab^	54.72 ± 0.28 ^a^	63.97 ± 3.66 ^b^	54.62 ± 0.28 ^a^	63.02 ± 3.32 ^b^	54.41 ± 0.03 ^a^	64.50 ± 1.99 ^b^
His	18.64 ± 1.75 ^ab^	17.91 ± 1.28 ^a^	22.41 ± 0.31 ^ab^	21.23 ± 0.29 ^b^	23.09 ± 0.07 ^ab^	19.09 ± 2.56 ^ab^	22.75 ± 0.44 ^ab^	20.75 ± 2.09 ^ab^	23.09 ± 0.35 ^ab^	21.23 ± 1.17 ^b^	23.10 ± 0.26 ^ab^	21.66 ± 0.80 ^b^
Arg	48.82 ± 4.01 ^ab^	45.42 ± 3.05 ^a^	52.22 ± 0.09 ^ab^	52.18 ± 0.68 ^b^	52.53 ± 0.08 ^ab^	48.80 ± 4.43 ^ab^	52.37 ± 0.27 ^ab^	52.13 ± 3.54 ^b^	52.47 ± 0.15 ^ab^	52.25 ± 2.56 ^b^	52.53 ± 0.18 ^ab^	53.43 ± 1.50 ^b^

The values in the table represent the average value of three measurements ± the standard deviation. Different lowercase letters in the same row indicate significant differences (*p* < 0.05).

**Table 6:** Changes in free amino acid profiles (μg/g) of egg white during the 42-day SeMet supplementation period.

	C-7 d	Se-7 d	C-14 d	Se-14 d	C-21 d	Se-21 d	C-28 d	Se-28 d	C-35 d	Se-35 d	C-42 d	Se-42 d
Asp	0.04 ± 0.01 ^ab^	0.08 ± 0.00 ^c^	0.03 ± 0.00 ^ab^	0.05 ± 0.04 ^ab^	0.03 ± 0.00 ^ab^	0.02 ± 0.02 ^a^	0.03 ± 0.00 ^ab^	0.04 ± 0.01 ^ab^	0.03 ± 0.00 ^ab^	0.07 ± 0.02 ^bc^	0.03 ± 0.00 ^ab^	0.05 ± 0.01 ^ab^
Thr	0.03 ± 0.00 ^a^	0.04 ± 0.00 ^a^	0.04 ± 0.00 ^a^	0.06 ± 0.00 ^b^	0.04 ± 0.00 ^a^	0.07 ± 0.00 ^bc^	0.04 ± 0.00 ^a^	0.07 ± 0.00 ^bc^	0.04 ± 0.00 ^a^	0.07 ± 0.00 ^c^	0.04 ± 0.00 ^a^	0.07 ± 0.00 ^c^
Ser	0.06 ± 0.01 ^a^	0.11 ± 0.00 ^d^	0.06 ± 0.01 ^a^	0.17 ± 0.01 ^e^	0.06 ± 0.01 ^a^	0.10 ± 0.01 ^cd^	0.06 ± 0.01 ^a^	0.08 ± 0.00 ^b^	0.06 ± 0.01 ^a^	0.09 ± 0.02 ^bc^	0.06 ± 0.01 ^a^	0.06 ± 0.01 ^a^
Gln	0.13 ± 0.01 ^a^	0.18 ± 0.00 ^b^	0.14 ± 0.01 ^a^	0.20 ± 0.01 ^bc^	0.13 ± 0.01 ^a^	0.21 ± 0.01 ^c^	0.13 ± 0.01 ^a^	0.27 ± 0.01 ^d^	0.14 ± 0.01 ^a^	0.18 ± 0.01 ^bc^	0.14 ± 0.01 ^a^	0.14 ± 0.01 ^a^
Ala	0.03 ± 0.01 ^ab^	0.06 ± 0.00 ^ab^	0.04 ± 0.00 ^ab^	0.09 ± 0.00 ^a^	0.04 ± 0.00 ^ab^	0.08 ± 0.01 ^a^	0.04 ± 0.00 ^ab^	0.09 ± 0.00 ^c^	0.04 ± 0.00 ^ab^	0.06 ± 0.01 ^bc^	0.04 ± 0.00 ^ab^	0.09 ± 0.00 ^ab^
Val	0.16 ± 0.01 ^a^	0.16 ± 0.00 ^ab^	0.16 ± 0.01 ^a^	0.16 ± 0.01 ^a^	0.16 ± 0.01 ^a^	0.15 ± 0.02 ^ab^	0.16 ± 0.02 ^a^	0.19 ± 0.00 ^ab^	0.16 ± 0.02 ^a^	0.18 ± 0.02 ^bc^	0.16 ± 0.02 ^a^	0.17 ± 0.01 ^c^
Met	0.08 ± 0.01 ^a^	0.09 ± 0.00 ^ab^	0.08 ± 0.01 ^a^	0.07 ± 0.00 ^a^	0.08 ± 0.01 ^a^	0.09 ± 0.02 ^a^	0.08 ± 0.02 ^a^	0.09 ± 0.00 ^ab^	0.08 ± 0.01 ^a^	0.11 ± 0.01 ^ab^	0.08 ± 0.02 ^a^	0.12 ± 0.02 ^b^
Ile	0.12 ± 0.00 ^ab^	0.13 ± 0.00 ^ab^	0.12 ± 0.00 ^ab^	0.12 ± 0.03 ^ab^	0.11 ± 0.00 ^ab^	0.11 ± 0.01 ^a^	0.11 ± 0.00 ^ab^	0.12 ± 0.01 ^ab^	0.12 ± 0.00 ^ab^	0.13 ± 0.01 ^ab^	0.12 ± 0.00 ^ab^	0.14 ± 0.02 ^b^
Leu	0.48 ± 0.02 ^a^	0.49 ± 0.00 ^b^	0.49 ± 0.02 ^a^	0.49 ± 0.06 ^b^	0.48 ± 0.03 ^a^	0.44 ± 0.05 ^bc^	0.47 ± 0.02 ^a^	0.52 ± 0.02 ^c^	0.48 ± 0.02 ^a^	0.48 ± 0.10 ^b^	0.49 ± 0.03 ^a^	0.54 ± 0.05 ^c^
Tyr	0.17 ± 0.01 ^a^	0.21 ± 0.01 ^b^	0.18 ± 0.01 ^a^	0.22 ± 0.01 ^b^	0.18 ± 0.01 ^a^	0.25 ± 0.03 ^bc^	0.18 ± 0.01 ^a^	0.27 ± 0.01 ^bc^	0.18 ± 0.01 ^a^	0.22 ± 0.01 ^a^	0.18 ± 0.02 ^a^	0.26 ± 0.03 ^c^
Phe	0.54 ± 0.02 ^a^	0.64 ± 0.01 ^ab^	0.56 ± 0.01 ^a^	0.63 ± 0.03 ^ab^	0.55 ± 0.02 ^a^	0.65 ± 0.07 ^ab^	0.54 ± 0.02 ^a^	0.68 ± 0.04 ^b^	0.55 ± 0.02 ^a^	0.55 ± 0.01 ^ab^	0.56 ± 0.03 ^a^	0.73 ± 0.06 ^b^
Arg	0.09 ± 0.01 ^ab^	0.10 ± 0.01 ^a^	0.09 ± 0.01 ^ab^	0.09 ± 0.00 ^a^	0.09 ± 0.01 ^ab^	0.10 ± 0.01 ^a^	0.09 ± 0.01 ^ab^	0.10 ± 0.01 ^ab^	0.09 ± 0.01 ^ab^	0.10 ± 0.01 ^ab^	0.09 ± 0.01 ^ab^	0.10 ± 0.01 ^b^

The values in the table represent the average value of three measurements ± the standard deviation. Different lowercase letters in the same row indicate significant differences (*p* < 0.05).

**Table 7:** Changes in total amino acid profiles (mg/g) of egg yolk during the 42-day SeMet supplementation period.

	C-7 d	Se-7 d	C-14 d	Se-14 d	C-21 d	Se-21 d	C-28 d	Se-28 d	C-35 d	Se-35 d	C-42 d	Se-42 d
Asp	17.70 ± 1.19 ^a^	31.62 ± 5.63 ^c^	18.01 ± 1.19 ^a^	23.93 ± 1.94 ^b^	18.27 ± 1.29 ^a^	31.90 ± 1.31 ^c^	18.52 ± 1.19 ^a^	32.80 ± 1.82 ^c^	18.69 ± 1.19 ^a^	40.61 ± 1.89 ^d^	18.45 ± 1.30 ^a^	31.81 ± 3.20 ^c^
Thr	10.05 ± 0.47 ^a^	17.46 ± 3.25 ^c^	10.23 ± 0.47 ^a^	13.55 ± 0.03 ^b^	10.39 ± 0.52 ^a^	17.24 ± 0.74 ^c^	10.53 ± 0.47 ^a^	18.05 ± 0.99 ^c^	10.62 ± 0.47 ^a^	22.17 ± 1.04 ^d^	10.49 ± 0.53 ^a^	18.04 ± 1.62 ^c^
Ser	16.60 ± 0.70 ^a^	8.46 ± 5.64 ^c^	16.91 ± 0.70 ^a^	22.92 ± 0.27 ^b^	17.17 ± 0.79 ^a^	28.50 ± 1.26 ^c^	17.40 ± 0.70 ^a^	29.76 ± 1.63 ^c^	17.56 ± 0.70 ^a^	37.01 ± 1.72 ^d^	17.34 ± 0.80 ^a^	29.12 ± 2.39 ^c^
Glu	18.94 ± 0.03 ^a^	40.06 ± 11.43 ^c^	19.31 ± 0.03 ^a^	31.07 ± 1.50 ^b^	19.63 ± 0.09 ^a^	40.31 ± 1.70 ^c^	19.88 ± 0.03 ^a^	40.46 ± 2.30 ^c^	20.07 ± 0.03 ^a^	54.47 ± 2.34 ^d^	19.82 ± 0.09 ^a^	41.18 ± 5.56 ^c^
Gly	6.35 ± 0.08 ^a^	10.45 ± 1.90 ^bc^	6.48 ± 0.08 ^a^	8.96 ± 0.31 ^b^	6.58 ± 0.12 ^a^	10.19 ± 0.49 ^bc^	6.67 ± 0.08 ^a^	10.39 ± 0.58 ^bc^	6.73 ± 0.08 ^a^	8.92 ± 0.60 ^b^	6.64 ± 0.12 ^a^	10.70 ± 0.88 ^c^
Ala	11.81 ± 0.15 ^a^	18.52 ± 3.82 ^c^	12.04 ± 0.15 ^a^	16.02 ± 0.78 ^bc^	12.23 ± 0.21 ^a^	17.70 ± 0.88 ^bc^	12.39 ± 0.15 ^a^	18.73 ± 1.02 ^c^	12.50 ± 0.15 ^a^	15.01 ± 1.08 ^b^	12.35 ± 0.21 ^a^	18.59 ± 1.82 ^c^
Cys	4.95 ± 0.15 ^a^	5.68 ± 0.96 ^a^	5.05 ± 0.15 ^a^	5.50 ± 0.20 ^a^	5.12 ± 0.18 ^a^	5.88 ± 0.30 ^a^	5.19 ± 0.15 ^a^	6.13 ± 0.34 ^a^	5.24 ± 0.15 ^a^	5.03 ± 0.35 ^a^	5.17 ± 0.18 ^a^	5.64 ± 0.46 ^a^
Val	17.40 ± 0.89 ^a^	19.99 ± 2.66 ^ab^	17.71 ± 0.89 ^a^	18.86 ± 1.24 ^ab^	17.98 ± 0.98 ^a^	19.86 ± 1.03 ^ab^	18.22 ± 0.89 ^a^	20.88 ± 1.14 ^b^	18.39 ± 0.89 ^a^	17.51 ± 1.21 ^a^	18.16 ± 0.99 ^a^	19.46 ± 1.51 ^ab^
Met	6.39 ± 1.26 ^a^	8.84 ± 0.72 ^b^	6.47 ± 1.26 ^a^	6.45 ± 1.74 ^a^	6.54 ± 1.30 ^a^	7.88 ± 0.35 ^ab^	6.64 ± 1.26 ^a^	8.83 ± 0.45 ^b^	6.70 ± 1.26 ^a^	8.42 ± 0.51 ^b^	6.61 ± 1.31 ^a^	8.26 ± 1.20 ^ab^
Ile	16.59 ± 1.44 ^a^	18.64 ± 2.24 ^a^	16.86 ± 1.44 ^a^	16.42 ± 1.55 ^a^	17.11 ± 1.53 ^a^	18.26 ± 0.90 ^a^	17.35 ± 1.44 ^a^	18.88 ± 1.05 ^a^	17.50 ± 1.44 ^a^	18.85 ± 1.09 ^a^	17.28 ± 1.54 ^a^	17.70 ± 1.14 ^a^
Leu	28.28 ± 1.62 ^a^	32.45 ± 5.11 ^ab^	28.78 ± 1.62 ^a^	29.07 ± 2.85 ^ab^	29.21 ± 1.77 ^a^	32.48 ± 1.58 ^ab^	29.61 ± 1.62 ^a^	33.58 ± 1.87 ^ab^	29.88 ± 1.62 ^a^	33.81 ± 1.94 ^b^	29.50 ± 1.78 ^a^	31.42 ± 3.18 ^ab^
Tyr	15.11 ± 0.84 ^a^	15.02 ± 1.02 ^a^	15.44 ± 0.84 ^a^	15.29 ± 1.19 ^a^	15.71 ± 0.77 ^a^	14.97 ± 0.85 ^a^	15.90 ± 0.84 ^a^	14.64 ± 0.87 ^a^	16.06 ± 0.84 ^a^	15.41 ± 0.85 ^a^	15.86 ± 0.77 ^a^	15.94 ± 1.14 ^a^
Phe	14.53 ± 0.24 ^a^	14.03 ± 1.67 ^a^	14.83 ± 0.24 ^a^	13.67 ± 0.49 ^a^	15.08 ± 0.17 ^a^	14.73 ± 0.75 ^a^	15.27 ± 0.24 ^a^	15.18 ± 0.85 ^a^	15.42 ± 0.24 ^a^	15.41 ± 0.88 ^a^	15.23 ± 0.17 ^a^	13.72 ± 1.16 ^a^
Lys	24.06 ± 1.29 ^ab^	24.41 ± 3.01 ^ab^	24.49 ± 1.29 ^a^	23.94 ± 1.26 ^ab^	24.86 ± 1.42 ^a^	24.75 ± 1.31 ^ab^	25.20 ± 1.29 ^a^	25.10 ± 1.43 ^ab^	25.43 ± 1.29 ^a^	27.20 ± 1.45 ^b^	25.11 ± 1.43 ^a^	23.57 ± 1.83 ^a^
His	7.44 ± 0.49 ^a^	8.16 ± 0.56 ^ab^	7.57 ± 0.49 ^a^	7.49 ± 0.48 ^a^	7.68 ± 0.53 ^a^	8.10 ± 0.41 ^ab^	7.79 ± 0.49 ^a^	7.97 ± 0.47 ^ab^	7.86 ± 0.49 ^a^	8.71 ± 0.46 ^b^	7.76 ± 0.53 ^a^	7.84 ± 0.25 ^a^
Arg	21.75 ± 1.01 ^a^	23.44 ± 1.88 ^ab^	22.15 ± 1.01 ^a^	21.83 ± 1.23 ^a^	22.48 ± 1.13 ^a^	22.92 ± 1.19 ^ab^	22.79 ± 1.01 ^a^	23.85 ± 1.32 ^ab^	22.99 ± 1.01 ^a^	24.98 ± 1.38 ^b^	22.70 ± 1.14 ^a^	22.67 ± 1.15 ^ab^
Pro	9.47 ± 0.28 ^ab^	8.39 ± 2.76 ^a^	9.64 ± 0.28 ^ab^	9.83 ± 0.54 ^ab^	9.79 ± 0.33 ^ab^	10.42 ± 0.54 ^ab^	9.92 ± 0.28 ^ab^	10.76 ± 0.60 ^ab^	10.02 ± 0.28 ^ab^	11.65 ± 0.62 ^b^	9.89 ± 0.33 ^ab^	9.80 ± 0.85 ^ab^

The values in the table represent the average value of three measurements ± the standard deviation. Different lowercase letters in the same row indicate significant differences (*p* < 0.05).

**Table 8:** Changes in free amino acid profiles (μg/g) of egg yolk during the 42-day SeMet supplementation period.

	**C-7 d**	**Se-7 d**	**C-14 d**	**Se-14 d**	**C-21 d**	**Se-21 d**	**C-28 d**	**Se-28 d**	**C-35 d**	**Se-35 d**	**C-42 d**	**Se-42 d**
Asp	22.87 ± 2.10 ^a^	23.81 ± 2.32 ^ab^	23.18 ^±^ 2.33 ^a^	24.96 ± 2.13 ^ab^	22.56 ± 2.36 ^a^	26.87 ± 1.36 ^bc^	22.35 ± 2.22 ^a^	26.07 ± 1.45 ^ab^	22.69 ± 1.85 ^a^	26.09 ± 1.79 ^ab^	23.04 ± 1.51 ^a^	29.92 ± 1.61 ^c^
Thr	25.39 ± 3.56 ^a^	27.13 ± 2.55 ^a^	25.80 ^±^ 3.91 ^a^	28.03 ± 2.01 ^a^	24.99 ± 4.17 ^a^	28.96 ± 0.27 ^ab^	24.79 ± 3.75 ^a^	26.96 ± 1.04 ^a^	25.16 ± 3.35 ^a^	27.51 ± 0.69 ^a^	25.56 ± 2.99 ^a^	31.63 ± 1.70 ^b^
Ser	28.89 ± 4.94 ^a^	30.25 ± 2.91 ^a^	29.33 ^±^ 5.33 ^a^	32.12 ± 1.29 ^ab^	28.44 ± 5.49 ^a^	31.81 ± 1.35 ^ab^	28.23 ± 5.17 ^a^	29.76 ± 0.88 ^a^	28.68 ± 4.67 ^a^	31.04 ± 1.56 ^ab^	29.10 ± 4.19 ^a^	35.19 ± 1.89 ^b^
Glu	22.41 ± 3.74 ^a^	23.30 ± 2.31 ^a^	22.76 ^±^ 4.05 ^a^	25.77 ± 1.72 ^ab^	22.07 ± 4.19 ^a^	24.90 ± 0.68 ^ab^	21.90 ± 3.92 ^a^	23.82 ± 0.61 ^ab^	22.25 ± 3.53 ^a^	24.20 ± 0.80 ^ab^	22.57 ± 3.17 ^a^	27.25 ± 1.46 ^b^
Gln	54.31 ± 7.52 ^a^	60.63 ± 5.04 ^ab^	55.13 ^±^ 8.24 ^a^	60.86 ± 2.36 ^ab^	53.49 ± 8.58 ^a^	59.57 ± 4.46 ^ab^	53.07 ± 7.92 ^a^	58.01 ± 0.82 ^ab^	53.89 ± 7.01 ^a^	57.71 ± 0.72 ^ab^	54.69 ± 6.14 ^a^	64.86 ± 3.49 ^b^
Gly	10.92 ± 1.85 ^a^	10.84 ± 1.18 ^a^	11.10 ^±^ 2.01 ^a^	11.71 ± 0.83 ^a^	10.74 ± 2.11 ^a^	11.40 ± 0.83 ^a^	10.67 ± 1.94 ^a^	10.73 ± 0.65 ^a^	10.83 ± 1.76 ^a^	11.10 ± 0.48 ^a^	10.99 ± 1.59 ^a^	12.60 ± 0.68 ^a^
AlA	14.16 ± 2.09 ^a^	15.07 ± 2.16 ^ab^	14.36 ^±^ 2.26 ^a^	15.65 ± 0.85 ^ab^	13.96 ± 2.30 ^a^	15.81 ± 1.35 ^ab^	13.84 ± 2.18 ^a^	14.33 ± 0.50 ^a^	14.07 ± 1.94 ^a^	15.38 ± 1.17 ^ab^	14.27 ± 1.71 ^a^	17.15 ± 0.92 ^b^
VAl	24.64 ± 3.37 ^a^	26.80 ± 2.67 ^ab^	25.02 ^±^ 3.71 ^a^	28.11 ± 2.23 ^ab^	24.25 ± 3.96 ^a^	27.99 ± 1.18 ^ab^	24.06 ± 3.56 ^a^	26.14 ± 0.16 ^a^	24.42 ± 3.17 ^a^	26.72 ± 1.09 ^ab^	24.80 ± 2.82 ^a^	30.23 ± 1.62 ^b^
Cys	3.55 ± 0.69 ^a^	4.02 ± 0.49 ^ab^	3.62 ^±^ 0.73 ^a^	4.18 ± 0.43 ^abc^	3.49 ± 0.79 ^a^	4.66 ± 0.10 ^bc^	3.46 ± 0.71 ^a^	3.93 ± 0.25 ^a^	3.51 ± 0.67 ^a^	3.94 ± 0.24 ^a^	3.57 ± 0.64 ^a^	4.87 ± 0.26 ^c^
Met	11.37 ± 1.66 ^a^	11.79 ± 1.59 ^ab^	11.54 ^±^ 1.80 ^a^	13.05 ± 1.10 ^ab^	11.20 ± 1.86 ^a^	12.58 ± 0.70 ^ab^	11.11 ± 1.74 ^a^	11.67 ± 0.47 ^a^	11.29 ± 1.55 ^a^	12.02 ± 0.57 ^ab^	11.45 ± 1.36 ^a^	13.74 ± 0.74 ^b^
Ile	20.33 ± 2.94 ^a^	21.79 ± 2.62 ^ab^	20.65 ^±^ 3.22 ^a^	24.01 ± 2.37 ^b^	20.01 ± 3.38 ^a^	22.64 ± 1.78 ^ab^	19.86 ± 3.09 ^a^	21.17 ± 0.11 ^ab^	20.16 ± 2.76 ^a^	21.68 ± 0.33 ^ab^	20.47 ± 2.45 ^a^	24.06 ± 1.29 ^b^
Leu	39.01 ± 4.69 ^a^	42.41 ± 5.02 ^ab^	39.60 ^±^ 5.21 ^a^	45.63 ± 3.57 ^b^	38.42 ± 5.53 ^a^	43.87 ± 4.13 ^ab^	38.11 ± 4.97 ^a^	40.67 ± 0.95 ^ab^	38.68 ± 4.34 ^a^	41.83 ± 1.54 ^ab^	39.28 ± 3.75 ^a^	46.78 ± 2.51 ^b^
Tyr	29.28 ± 3.89 ^a^	31.98 ± 2.37 ^ab^	29.72 ^±^ 4.27 ^a^	33.01 ± 2.04 ^ab^	28.85 ± 4.44 ^a^	32.91 ± 1.24 ^ab^	28.61 ± 4.10 ^a^	30.81 ± 0.48 ^a^	29.06 ± 3.60 ^a^	32.12 ± 0.54 ^ab^	29.49 ± 3.14 ^a^	35.44 ± 1.90 ^b^
Phe	20.76 ± 2.89 ^a^	21.78 ± 1.97 ^ab^	21.07 ^±^ 3.17 ^a^	24.78 ± 2.15 ^b^	20.44 ± 3.33 ^a^	22.14 ± 1.82 ^ab^	20.28 ± 3.05 ^a^	21.45 ± 0.36 ^a^	20.59 ± 2.71 ^a^	21.19 ± 0.42 ^a^	20.90 ± 2.38 ^a^	23.78 ± 1.28 ^ab^
g-ABA	0.09 ± 0.00 ^bc^	0.10 ± 0.01 ^c^	0.09 ^±^ 0.00 ^bc^	0.07 ± 0.01 ^a^	0.09 ± 0.00 ^bc^	0.09 ± 0.00 ^b^	0.08 ± 0.00 ^bc^	0.09 ± 0.00 ^b^	0.09 ± 0.01 ^bc^	0.09 ± 0.01 ^bc^	0.09 ± 0.01 ^bc^	0.08 ± 0.00 ^a^
Lys	45.08 ± 6.93 ^a^	49.19 ± 6.09 ^ab^	45.77 ^±^ 7.55 ^a^	51.45 ± 3.31 ^ab^	44.38 ± 7.87 ^a^	50.49 ± 2.69 ^ab^	44.04 ± 7.28 ^a^	46.21 ± 1.71 ^a^	44.72 ± 6.53 ^a^	49.14 ± 2.05 ^ab^	45.39 ± 5.81 ^a^	54.31 ± 2.92 ^b^
His	9.65 ± 1.93 ^a^	10.25 ± 1.25 ^a^	9.80 ^±^ 2.07 ^a^	11.30 ± 0.83 ^a^	9.50 ± 2.12 ^a^	10.53 ± 0.34 ^a^	9.43 ± 2.01 ^a^	9.80 ± 0.26 ^a^	9.59 ± 1.84 ^a^	10.35 ± 0.47 ^a^	9.72 ± 1.68 ^a^	11.40 ± 0.61 ^a^
Arg	40.88 ± 5.82 ^a^	44.43 ± 5.97 ^a^	41.51 ^±^ 6.37 ^a^	47.25 ± 3.79 ^a^	40.26 ± 6.67 ^a^	44.97 ± 3.80 ^a^	39.94 ± 6.13 ^a^	41.20 ± 1.09 ^a^	40.56 ± 5.45 ^a^	43.44 ± 2.06 ^a^	41.17 ± 4.81 ^a^	47.53 ± 2.55 ^a^

The values in the table represent the average value of three measurements ± the standard deviation. Different lowercase letters in the same row indicate significant differences (*p* < 0.05).

**Table 9:** Changes in fatty acid composition (mg/g) of egg yolk during the 42-day SeMet supplementation period.

	C-7 d	Se-7 d	C-14 d	Se-14 d	C-21 d	Se-21 d	C-28 d	Se-28 d	C-35 d	Se-35 d	C-42 d	Se-42 d
C8:0	0.0018 ± 0.00 ^a^	0.0018 ± 0.00 ^a^	0.0018 ± 0.00 ^a^	0.0019 ± 0.00 ^a^	0.0019 ± 0.00 ^a^	0.0024 ± 0.0002 ^a^	0.0019 ± 0.00 ^a^	0.0023 ± 0.0002 ^a^	0.0019 ± 0.00 ^a^	0.0023 ± 0.0002 ^a^	0.0019 ± 0.00 ^a^	0.0022 ± 0.0002 ^a^
C10:0	0.0010 ± 0.00 ^a^	0.0031 ± 0.00 ^a^	0.0010 ± 0.00 ^a^	0.0041 ± 0.00 ^ab^	0.0010 ± 0.00 ^a^	0.0045 ± 0.0003 ^c^	0.0010 ± 0.00 ^a^	0.0053 ± 0.0004 ^ab^	0.0010 ± 0.00 ^a^	0.0089 ± 0.0007 ^b^	0.0010 ± 0.00 ^a^	0.0096 ± 0.0007 ^d^
C12:0	0.0029 ± 0.00 ^a^	0.0036 ± 0.00 ^a^	0.0029 ± 0.00 ^a^	0.0053 ± 0.00 ^b^	0.0030 ± 0.00 ^a^	0.0053 ± 0.0004 ^b^	0.0029 ± 0.00 ^a^	0.0051 ± 0.0004 ^b^	0.0030 ± 0.00 ^a^	0.0062 ± 0.0005 ^b^	0.0030 ± 0.00 ^a^	0.0077 ± 0.0006 ^c^
C14:1	0.08 ± 0.01 ^a^	0.13 ± 0.01 ^a^	0.08 ± 0.01 ^a^	0.11 ± 0.01 ^a^	0.09 ± 0.01 ^a^	0.10 ± 0.01 ^a^	0.08 ± 0.01 ^a^	0.11 ± 0.01 ^a^	0.09 ± 0.01 ^a^	0.16 ± 0.01 ^b^	0.09 ± 0.01 ^a^	0.25 ± 0.02 ^c^
C14:0	0.34 ± 0.03 ^a^	0.37 ± 0.03 ^a^	0.35 ± 0.03 ^a^	0.40 ± 0.03 ^a^	0.35 ± 0.03 ^a^	0.39 ± 0.03 ^a^	0.35 ± 0.03 ^a^	0.36 ± 0.03 ^a^	0.35 ± 0.03 ^a^	0.41 ± 0.03 ^a^	0.35 ± 0.03 ^a^	0.68 ± 0.05 ^b^
C15:0	0.03 ± 0.00 ^a^	0.04 ± 0.00 ^ab^	0.03 ± 0.00 ^a^	0.05 ± 0.00 ^b^	0.03 ± 0.00 ^a^	0.04 ± 0.00 ^b^	0.03 ± 0.00 ^a^	0.05 ± 0.00 ^b^	0.03 ± 0.00 ^a^	0.06 ± 0.00 ^c^	0.03 ± 0.00 ^a^	0.07 ± 0.01 ^d^
C16:1	12.31 ± 0.96 ^a^	13.05 ± 1.01 ^a^	12.43 ± 1.11 ^a^	12.51 ± 0.97 ^a^	12.55 ± 1.12 ^a^	13.55 ± 1.05 ^a^	12.49 ± 1.11 ^a^	13.15 ± 1.02 ^a^	12.60 ± 1.12 ^a^	14.45 ± 1.12 ^a^	12.66 ± 1.13 ^a^	21.38 ± 1.66 ^b^
C16:0	16.70 ± 1.30 ^a^	16.96 ± 1.32 ^a^	16.86 ± 1.50 ^a^	17.28 ± 1.34 ^a^	17.02 ± 1.52 ^a^	17.64 ± 1.37 ^a^	16.93 ± 1.51 ^a^	17.82 ± 1.38 ^a^	17.09 ± 1.52 ^a^	20.84 ± 1.62 ^a^	17.17 ± 1.53 ^a^	23.95 ± 1.86 ^a^
C17:1	0.27 ± 0.02 ^a^	0.35 ± 0.03 ^a^	0.27 ± 0.02 ^a^	0.45 ± 0.03 ^b^	0.27 ± 0.02 ^a^	0.48 ± 0.04 ^b^	0.27 ± 0.02 ^a^	0.51 ± 0.04 ^b^	0.27 ± 0.02 ^a^	0.60 ± 0.05 ^c^	0.28 ± 0.02 ^a^	0.67 ± 0.05 ^c^
C18:2	24.56 ± 1.91 ^a^	29.48 ± 2.29 ^ab^	24.79 ± 2.21 ^a^	30.44 ± 2.36 ^ab^	25.04 ± 2.23 ^a^	29.88 ± 2.32 ^ab^	24.91 ± 2.22 ^a^	32.82 ± 2.55 ^b^	25.14 ± 2.24 ^a^	38.64 ± 3.00 ^c^	25.26 ± 2.25 ^a^	41.75 ± 3.24 ^c^
C18:1	30.65 ± 2.38 ^a^	29.64 ± 2.30 ^a^	30.95 ± 2.76 ^a^	31.10 ± 2.42 ^a^	31.25 ± 2.79 ^a^	29.99 ± 2.33 ^a^	31.09 ± 2.77 ^a^	32.22 ± 2.50 ^a^	31.38 ± 2.80 ^a^	39.31 ± 3.05 ^b^	31.53 ± 2.81 ^a^	47.55 ± 3.69 ^c^
C18:1	6.68 ± 0.52 ^ab^	6.14 ± 0.48 ^a^	6.74 ± 0.60 ^ab^	7.32 ± 0.57 ^bc^	6.81 ± 0.61 ^ab^	7.54 ± 0.59 ^bc^	6.77 ± 0.60 ^ab^	7.48 ± 0.58 ^bc^	6.84 ± 0.61 ^ab^	8.03 ± 0.62 ^c^	6.87 ± 0.61 ^ab^	12.24 ± 0.95 ^d^
C18:0	10.54 ± 0.82 ^a^	10.52 ± 0.82 ^a^	10.64 ± 0.95 ^a^	12.82 ± 1.00 ^b^	10.75 ± 0.96 ^a^	12.30 ± 0.96 ^ab^	10.69 ± 0.95 ^a^	12.74 ± 0.99 ^b^	10.79 ± 0.96 ^a^	17.89 ± 1.39 ^c^	10.84 ± 0.97 ^a^	21.87 ± 1.70 ^d^
C20:4	0.16 ± 0.01 ^a^	0.24 ± 0.02 ^b^	0.16 ± 0.01 ^a^	0.68 ± 0.05 ^c^	0.16 ± 0.01 ^a^	0.65 ± 0.05 ^c^	0.16 ± 0.01 ^a^	0.64 ± 0.05 ^c^	0.16 ± 0.01 ^a^	0.64 ± 0.05 ^c^	0.16 ± 0.01 ^a^	0.77 ± 0.06 ^d^
C20:2	1.93 ± 0.15 ^a^	2.07 ± 0.16 ^a^	1.95 ± 0.17 ^a^	2.86 ± 0.22 ^b^	1.97 ± 0.18 ^a^	2.87 ± 0.22 ^b^	1.96 ± 0.17 ^a^	2.95 ± 0.23 ^b^	1.98 ± 0.18 ^a^	5.12 ± 0.40 ^c^	1.99 ± 0.18 ^a^	6.33 ± 0.49 ^d^
C20:0	0.02 ± 0.00 ^a^	0.04 ± 0.00 ^b^	0.02 ± 0.00 ^a^	0.04 ± 0.00 ^b^	0.02 ± 0.00 ^a^	0.11 ± 0.01 ^c^	0.02 ± 0.00 ^a^	0.11 ± 0.01 ^c^	0.02 ± 0.00 ^a^	0.17 ± 0.01 ^d^	0.02 ± 0.00 ^a^	0.20 ± 0.02 ^e^
C22:6	0.01 ± 0.00 ^a^	0.02 ± 0.00 ^b^	0.01 ± 0.00 ^a^	0.02 ± 0.00 ^b^	0.01 ± 0.00 ^a^	0.02 ± 0.00 ^b^	0.01 ± 0.00 ^a^	0.03 ± 0.00 ^c^	0.01 ± 0.00 ^a^	0.05 ± 0.00 ^d^	0.01 ± 0.00 ^a^	0.05 ± 0.00 ^e^

The values in the table represent the average value of three measurements ± the standard deviation. Different lowercase letters in the same row indicate significant differences (*p* < 0.05).

## Data Availability

The original contributions presented in this study are included in the article and Supplementary Material. Further inquiries can be directed to the corresponding author.

## Supplementary Materials

The following supporting information can be downloaded at: https://www.mdpi.com/article/10.3390/foods15162859/s1. Table S1: Effect of SeMet on production performance.
