# Supplementary material for: Regulation of Egg Quality by Methionine Selenium: Physicochemical and Structural Characteristics of EggShell, Egg Yolk, and Egg White
Source: Foods. 2026 Aug 17;15(16):2859. doi: 10.3390/foods15162859 (PMC13512702; doi:10.3390/foods15162859)
Supplement: Supplementary file 1 [file foods-15-02859-s001.zip › foods-4383805-supplementary.pdf]

Table S1 Effect of SeMet on production performance

group	laying rate（%）	defective egg rate（%）	proportion of qualified eggs（%）	average egg weight （g）
C	0.83 ± 0.01^{b}	0.03 ± 0.01^{a}	0.88 ± 0.05^{b}	61.52 ± 1.18^{a}
Se	0.88 ± 0.01^{a}	0.01 ± 0.01^{b}	0.93 ± 0.02^{a}	63.11 ± 1.84^{a}

The values in the table represent the average value of measurements ± the standard deviation. Different lowercase letters in the same row indicate significant differences ($P < 0.05$).
